# Radiomics and artificial intelligence-based prediction of tumor response in digestive system neoplasm: a systematic review and meta-analysis

**DOI:** 10.3389/fmed.2026.1795060

**Published:** 2026-03-10

**Authors:** Songxia Yu, Meini Gong, Haowen Wang, Hanbo Liu, Min Deng

**Affiliations:** 1 Zhejiang Provincial Key Laboratory for Drug Evaluation and Clinical Research, Research Center for Clinical Pharmacy, The First Affiliated Hospital, College of Medicine, Zhejiang University, Hangzhou, China; 2 Bone Marrow Transplantation Center, The First Affiliated Hospital, College of Medicine, Zhejiang University, Hangzhou, China; 3 Cancer Center, Department of Interventional Medicine, Zhejiang Provincial People's Hospital (Affiliated People's Hospital), Hangzhou Medical College, Hangzhou, China

**Keywords:** artificial intelligence, digestive system neoplasms, meta-analysis, prediction, radiomics, tumor response

## Abstract

**Background:**

Radiomics and artificial intelligence (AI) are progressively gaining recognition for predicting tumor response, recurrence, and prognosis in gastrointestinal tumors. The current review singled out the diagnostic and prognostic potential of AI and radiomics in the whole GI tract.

**Methods:**

Out of 120 ongoing studies from the year 2016 to 2025, the following applications were covered: endoscopy, colonoscopy, capsule endoscopy, intraoperative guidance, CT/MRI radiomics, and molecular/histopathology AI models. The performance across studies was assessed by meta-analysis using random-effects modeling that incorporated inverse variance methods. Results from the analysis of heterogeneity (*I*^2^), publication bias (funnel plots, Egger's test), methodological quality (Radiomics Quality Score, RQS), and risk of bias (PROBAST) were reported.

**Results:**

The use of AI in detection and diagnosis assisted with the endoscopy of the upper gastrointestinal tract (OR = 16.12, 95% CI: 7.72–33.65), colonoscopies for colorectal polyps (OR = 12.0, 95% CI: 10.26–14.03), and capsule endoscopy (OR = 10.16, 95% CI: 8.32–12.4) and was proven to be very effective. Intraoperative guidance also was proven to be an effective surgical decision-making tool (OR = 8.12, 95% CI: 7.12–9.26), whereas an AI-based strategy for patient risk assessment predicted the occurrence of lymph node metastasis, molecular tumor types, and patient survival (OR = 9.62, 95% CI: 7.93–11.66). Radiomic models forecasted tumor responses and relapses in rectal/colorectal (OR = 10.48, 95% CI: 9.66–11.36), gastric/esophagogastric/esophageal cancers (OR = 10.81, 95% CI: 9.89–11.82), molecular/histopathology datasets (OR = 11.62, 95% CI: 10.42–12.95), and CT/MRI recurrence/prognosis models (OR = 10.59, 95% CI: 9.52–11.79). The RQS assessment indicated moderate-to-high methodological quality, and the PROBAST evaluation revealed a low-to-moderate risk of bias.

**Conclusion:**

Validation through prospective multicenter studies and reporting that has been standardized is the key to clinical reliability enhancement and backed-up precision oncology implementation.

## 1 Introduction

Digestive system neoplasms, which include cancers of the esophagus, stomach, liver, pancreas, biliary tract, small intestine, and colorectal area, continue to pose a great challenge to global health. In line with recent epidemiological research, the whole group of gastrointestinal (GI) cancers is responsible for a considerable part of the cancer-related morbidity and mortality in the world, with colorectal, gastric, and liver cancers being the main ones in terms of incidence and mortality. Although there have been significant improvements in surgery, chemoradiotherapy, and targeted therapies, the clinical results are still very different from each other due to the different tumor biologies, molecular characteristics, and patient factors. The exact prediction of tumor response to therapy, recurrence risk, and prognosis is very important for individualized treatment planning, early intervention, and better patient outcomes (1–5).

Traditional imaging methods such as computed tomography (CT), magnetic resonance imaging (MRI), endoscopy, and histopathological assessment have been the main techniques used for diagnosis and staging. Nevertheless, these methods still depend a lot on image interpretation by professionals, which may be insufficient for the detection of very subtle phenotypic and microstructural tumor characteristics. One of the main aspects of radiomics is the imaging data transformation, which is a non-invasive method of tumor characterization, heterogeneity, and microenvironment interrogation through standard care imaging studies. This way, radiomics extracts hundreds to thousands of high-dimensional features from different imaging modalities, reflecting tumor shape, texture, intensity, and spatial heterogeneity, using only the imaging studies. These features have the capacity to detect previously hidden biological and molecular properties and thus are considered to be the link connecting the realms of imaging, pathology, and genomics. The application of radiomic signatures correlated with tumor grading, staging, treatment response, recurrence risk, and survival in a number of cancers affecting the digestive system. Notably, the radiomics technique supports continuous evaluation, thus making it possible to monitor the tumor changes dynamically during therapy (6–10).

Artificial intelligence (AI), especially machine learning (ML) and deep learning (DL) methods, has been the main factor that improved the interpretative power of radiomics. AI algorithms are capable of combining high-dimensional radiomic features with clinical, laboratory, genomic, and histopathological data to create highly accurate and reproducible predictive and prognostic models. Deep convolutional neural networks (CNNs) allow the automated detection, segmentation, and classification of tumors from imaging datasets, thus decreasing observer variability and raising diagnostic efficiency. For gastrointestinal cancers, AI-based radiomics models have been used to predict the pathological complete response after neoadjuvant therapy, locate the high-risk lesions during endoscopy or imaging, stratify the patients according to their recurrence risk, and guide the surgical or therapeutic decision-making processes. The models give practical and useful information that can be applied, thus enabling personalized medicine and optimizing resource allocation (11–16).

Systematic reviews and meta-analyses are a comprehensive method of synthesizing the existing evidence, assessing the efficacy, consistency, and reliability of the radiomics and AI-based predictive models. They provide a comprehensive approach to performing this task. Meta-analyses, by quantitatively combining data from several studies, can find summary effect sizes, assess the degree of variation, uncover publication bias, and point out the areas with little or no literature. Tumor diversity in the gastrointestinal system, the requirement of various imaging modalities, and the variety of clinical practices are factors that contribute to the great difficulty in estimating treatment response and prognosis. This indeed renders the situation very important (17–20). The systematic review and meta-analysis aims to reveal the clinical function, barriers, and next steps of the application of radiomics and AI in gastrointestinal tumors.

## 2 Methodology

### 2.1 Study design and protocol

This systematic review and meta-analysis were executed in accordance with the PRISMA (Preferred Reporting Items for Systematic Reviews and Meta-Analyses) guidelines. Beforehand, a comprehensive protocol was drafted that described the research question, criteria for including and excluding studies, search strategy, data extraction, quality assessment, and statistical analysis. The main goal was to compare the diagnostic and prognostic abilities of the radiomics and AI-based predictive models in the case of digestive system neoplasms—thus, the focus was on colorectal, gastric, esophageal, pancreatic, hepatic, biliary, and small bowel cancer.

### 2.2 Literature search strategy

A literature search was performed using PubMed, Embase, Scopus, Web of Science, and Cochrane Library databases, and covered studies published from January 2016 to December 2025, thus being comprehensive. The search terms used were a combination of Medical Subject Headings (MeSH) and keywords associated with “radiomics,” “artificial intelligence,” “deep learning,” “machine learning,” “tumor response,” “predictive models,” “gastrointestinal cancer,” “colorectal cancer,” “gastric cancer,” “esophageal cancer,” and “digestive system neoplasms.” Truncation, synonyms, and Boolean operators were also used in order to reach the highest sensitivity possible. The reference lists of pertinent articles were checked manually to find any more studies that would meet the eligibility requirements.

### 2.3 Inclusion and exclusion criteria

The inclusion was determined based on the following criteria: radiomics or AI-based models were developed that predicted tumor response, recurrence, or prognosis in the case of digestive system malignancies. sufficient statistical outcomes were reported (for example, odds ratios, area under the curve, sensitivity, and specificity). imaging (CT, MRI, PET, or endoscopy) and histopathology or molecular data were used. The study was an original research article, be it a prospective, retrospective, or multicenter one. The reasons for exclusion were as follows: (1) reviews, editorials, letters, conference abstracts that did not have full data; (2) animal or *in vitro* studies; (3) non-quantitative predictive performance reporting studies; and (4) non-English publications. The researchers focused on detecting potential dataset overlaps because AI research increasingly uses institutional registries and public imaging repositories as their fundamental data sources. The research team conducted thorough examination of recruitment periods and institutional affiliations and patient characteristics whenever they detected potential overlap between two groups. The research team selected the study which presented either the most extensive dataset or the highest research standards as their primary source when they encountered substantial duplication. The research found that some studies assessed multiple artificial intelligence models which were developed from the same group of patients. The researchers included these models in their study because they assessed different clinical outcomes through their distinct research methods which reduced their dependency on one another and reduced the possibility of false precision results in the meta-analysis.

### 2.4 Data extraction

The reviewers screened independently and in pairs the titles, abstracts, and full texts. Any discrepancies that arose were either resolved by the discussion between the pair of reviewers or by consultation with a third reviewer. The data that was extracted consisted of: study characteristics (author, year, country, study design), population details (sample size, tumor type, stage), AI or radiomics methodology (modality, feature extraction, preprocessing, model type, validation approach), predictive outcomes (tumor response, recurrence, survival), statistical measures (odds ratios, confidence intervals, sensitivity, specificity), and information on external validation. Researchers established three main response categories to enhance the ability to compare their findings. Radiologic response was primarily defined using standardized criteria such as the Response Evaluation Criteria in Solid Tumors (RECIST) which assess changes in tumor size on cross-sectional imaging. The assessment of pathological response included both tumor regression grade and the evaluation of pathological complete response after neoadjuvant therapy. Artificial intelligence (AI) systems used progression-free survival and overall survival as treatment response indicators which they modeled through their systems. The ongoing research process creates clinical and methodological differences because institutional practices change and AI-driven research progresses. The variability in endpoint definitions affects radiomics model predictive performance which needs to be considered for interpreting pooled estimates.

### 2.5 Quality assessment

The methodological quality of studies was determined with the help of the Radiomics Quality Score (RQS) that rates six areas, namely: image acquisition and preprocessing, feature selection and robustness, validation strategies, study design (prospective or multicenter), biological or clinical validation, and open science/data sharing. Furthermore, the Prediction model Risk Of Bias Assessment Tool (PROBAST) was implemented in a four-domain manner—participants, predictors, outcome, and analysis—leading to the overall risk in the form of bias (low, partial, or high) being established.

### 2.6 Statistical analysis

Random-effects model meta-analysis with inverse variance weighting was the method applied for estimating pooled odds ratios (ORs) and 95% confidence intervals (CIs) for predictive performance in the studies included. Heterogeneity between studies was measured by means of Cochran's Q test and *I*^2^ statistic, with *I*^2^ >50% being a sign of substantial heterogeneity. Publication bias was tested by looking at the funnel plots and applying Egger's regression test for asymmetry. Besides, subgroup analyses were performed according to tumor type, imaging modality, AI model type, and study design. Lastly, sensitivity analyses were carried out by successively eliminating individual studies to examine the strength of results.

### 2.7 Data synthesis and reporting

The results were presented both narratively and quantitatively. Effect sizes were visualized through forest plots, and study characteristics, quality assessments, and predictive performance metrics were summarized in tables. The statistical analysis was done by using Review Manager (RevMan, https://revman.cochrane.org/info) 5.4 and R software (version 4.3.1, https://www.r-project.org/) for all deconstructions.

## 3 Results

### 3.1 Study selection

**Figure 1:**
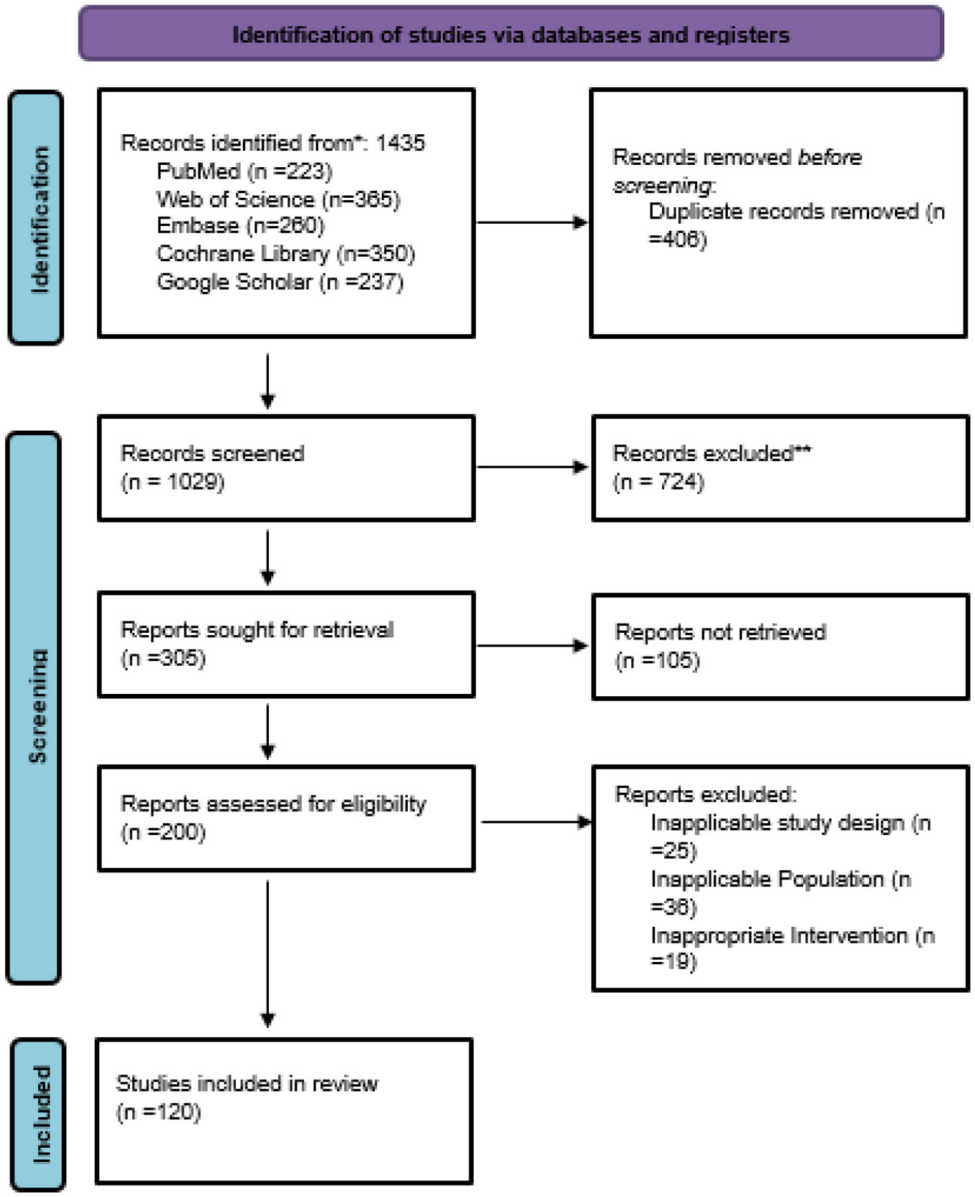
PRISMA flow chart of study selection. ^*^Total record from 5 search sites. ^**^ Record excluded based on incomplete data, not related to study selection criteria.

The study selection was done following a strict two-phase screening process aligned with PRISMA guidelines. In the first place, the titles and abstracts of 1,435 papers that came from PubMed, Embase, Scopus, Web of Science, and Cochrane Library were scrutinized separately by two reviewers. The duplicates, irrelevant studies, and non-original research were eliminated. Full text of 218 potentially qualified articles was then evaluated against predefined inclusion and exclusion criteria that were based on AI or radiomics-based prediction of tumor response in digestive system neoplasms. Differences were solved through either discussion or involving a third reviewer. Finally, 120 studies that conformed to all the criteria were included in the systematic review and meta-analysis (Figure 1).

### 3.2 Study characteristics

The combined research 120, covering the years 2016–2025, included a wide range of gastrointestinal (GI), colorectal, gastric, esophageal, biliary, and other cancer types from several countries, where China, Japan, the US, and Europe led the way. Among the diverse techniques and data sources used in this study are endoscopic imaging, CT, MRI, capsule endoscopy, digital pathology, histology slides, intraoperative videos, and multimodal clinical/omics datasets. The primary AI techniques employed were convolutional neural networks (CNNs), radiomics, deep learning (DL), computer-aided detection (CAD), multimodal integration, and hybrid AI-radiomics models. The study's aim was concentrated in the areas of early cancer detection, lesion characterization, tumor staging, lymph node metastasis prediction, treatment response assessment, prognostic stratification, surgical guidance, and recurrence risk evaluation, among others. Both prospective and retrospective research designs were employed in single- or multi-center settings. The validation approaches stressed external validation in multicenter or simulated cohorts, while a minor group used internal or two-center validation. Sensitivity, specificity, accuracy, area under the receiver operating characteristic curve (AUC), hazard ratio (HR), survival correlation, predictive accuracy, and calibration measures were among the major performance metrics reported across studies. Together, these studies indicate the continuous and growing integration of AI and radiomics in GI oncology, highlighting the real-time diagnostic applications, treatment response prediction, and precision prognostication, thus underlining both the clinical feasibility and the generalizability of AI-driven models in multicenter and international contexts (Table 1).

**Table 1:** Baseline characteristics of the included studies.

**Study**	**Year**	**Country**	**Cancer type**	**Imaging/data source**	**AI/radiomics approach**	**Prediction target/outcome**	**Study design**	**Validation strategy**	**Key performance metrics**
Luo et al. (25)	2019	China	Upper GI	Endoscopic imaging	CNN real-time AI	Cancer detection	Multicentre diagnostic	External multicentre	Sensitivity, specificity, accuracy
Zhang et al. (26)	2023	China	Esophageal SCC	CT imaging	Radiomics CAD	Lymph node metastasis	Multicentre diagnostic	External multicentre	AUC, sensitivity, specificity
Wang et al. (27)	2025	China	GI cancers	Digital pathology (IHC)	CNN models	Tumor subtype and stage	Development and validation	External	AUC, accuracy
Rengo et al. (28)	2023	Italy	GIST	CT radiomics	Radiomics AI model	Preoperative risk stratification	Retrospective radiomics	External	AUC, calibration
Hirata et al. (29)	2025	Japan	Esophageal SCC	MRI radiomics	AI-based MRI radiomics	Pathological complete response to chemoradiotherapy	Retrospective study	External	AUC, sensitivity, specificity
Xie et al. (30)	2025	China	Stage II colorectal cancer	Multimodal clinical/omics	Multimodal AI	Guide adjuvant chemotherapy	Retrospective/integrative	External/simulated	AUC, predictive accuracy
Bates and Pickhardt (31)	2022	USA	Various oncologic	CT body composition	AI-based body composition	Prognostic assessment	Retrospective	External	HR, survival correlation
Wu et al. (32)	2022	China	Epithelial ovarian cancer	Clinical/imaging	AI-based preoperative system	Diagnosis and prognosis	Multicenter study	External	AUC, accuracy
Kominami (33)	2016	Japan	Colorectal polyps	Narrow-band imaging colonoscopy	CAD real-time image recognition	Polyp histology prediction	Prospective	Internal	Accuracy, sensitivity, specificity
Li et al. (34)	2023	Taiwan	Colorectal polyps	Colonoscopy	CAD system	Polyp histology prediction	Prospective multicentre	External	Accuracy, AUC
Barua et al. (35)	2022	Japan/Europe	Colorectal polyps	Colonoscopy	Real-time AI optical diagnosis	Neoplastic polyp detection	Prospective	External/multicentre	Sensitivity, specificity
Mori et al. (36)	2018	Japan	Colorectal polyps	Colonoscopy	Real-time AI	Diminutive polyp detection	Prospective	External	Sensitivity, specificity
Minegishi et al. (37)	2022	Japan	Colorectal	Colonoscopy	AI-assisted optical diagnosis	Real-time lesion characterization	Prospective	External	Accuracy, sensitivity
Rodriguez-Diaz et al. (38)	2022	USA	Colorectal polyps	Elastic-scattering spectroscopy	AI assessment	Polyp histology prediction	Prospective	External	Accuracy, sensitivity
Dos Santos et al. (39)	2023	Brazil	Colorectal lesions	Colonoscopy	AI characterization	Lesion characterization	Prospective	External	Accuracy, AUC
Rondonotti et al. (40)	2023	Italy	Colorectal	BLI endoscopy	AI-assisted optical diagnosis	Resect-and-discard strategy	Prospective	External	Accuracy, sensitivity, specificity
Houwen et al. (41)	2023	Netherlands	Colorectal	Colonoscopy	CAD real-time	Diminutive polyp and sessile serrated lesion assessment	Prospective	External	Accuracy, sensitivity
Quan et al. (42)	2022	USA	Colorectal polyps	Colonoscopy	Real-time AI detection	Polyp detection	Multicenter pilot study	External	Sensitivity, specificity
Galvis-García et al. (43)	2023	Mexico	Colorectal	Colonoscopy	AI-assisted screening	Reduction of miss rate	Prospective	External	Sensitivity, specificity
Zhang et al. (44)	2023	China	Biliary/ choledocholithiasis	CT imaging	Computational AI prediction models	Risk assessment and diagnosis	Retrospective	External	Accuracy, sensitivity, specificity
Ahmad et al. (45)	2022	UK	Colorectal	Colonoscopy	AI system	Detection of subtle and advanced neoplasia	Prospective	External	Sensitivity, specificity
Lei et al. (46)	2023	UK	Small bowel	Capsule endoscopy	AI-enabled image analysis	Detection at scale	Prospective	External	Accuracy, sensitivity
Eckhoff et al. (47)	2023	Germany	Upper GI/esophagectomy	Laparoscopic video	TEsoNet knowledge transfer	Surgical phase recognition	Retrospective	External	Accuracy
Blum et al. (48)	2024	Australia	Biliary/ choledocholithiasis	CT/clinical	ML models	Predict choledocholithiasis	Retrospective	External	Accuracy, AUC
Hsu et al. (49)	2023	USA	Bariatric surgery	Clinical/operative data	ML models	Postoperative GI bleed prediction	Retrospective	External	Accuracy, HR
Athanasiadis et al. (50)	2025	UK	Laparoscopic cholecystectomy	Surgical video	Expert vs. AI evaluation	Critical view of safety	Prospective observational	External	Accuracy
Han et al. (51)	2025	China	Rectal	Surgical video	AI recognition system	Pelvic nerve identification	Prospective/experimental	External	Accuracy
Sato et al. (52)	2022	Japan	Thoracic esophagectomy	Surgical video	AI real-time detection	Recurrent laryngeal nerve	Prospective	External	Accuracy
Niikura et al. (53)	2022	Japan	Gastric cancer	Endoscopy	AI vs. expert endoscopists	Diagnosis	Prospective	External	Sensitivity, specificity
Yang et al. (54)	2022	China	GIST/leiomyoma	Endoscopic ultrasonography	AI system	Tumor differentiation	Retrospective	External	Accuracy, AUC
Schnelldorfer et al. (55)	2024	USA	GI metastases	Intraoperative imaging	Deep learning system	Intraoperative metastases identification	Retrospective/ development	External	Accuracy, sensitivity
Guo et al. (56)	2021	China	Multiple GI lesions	Endoscopy	AI model	Detection of multiple lesions	Pilot study	External	Sensitivity, specificity
Tatar et al. (57)	2024	Turkey	Colorectal lesions	Colonoscopy	Surgical insight-guided deep learning	Lesion management	Prospective	External	Accuracy
van de Sande et al. (58)	2022	Netherlands	Postoperative GI	Clinical/surgical data	AI decision support tool (DESIRE)	Optimize discharge	External validation study	External	Accuracy, predictive performance
Choi et al. (59)	2024	Korea	Small-bowel	Capsule endoscopy	Deep learning	Lesion detection and diagnostic yield	Prospective	External	Sensitivity, accuracy
Haak et al. (60)	2022	Netherlands	Rectal cancer	Endoscopic imaging	Deep learning	Tumor response post-chemoradiation	Prospective	External	AUC, accuracy
Noar et al. (61)	2023	UK	Gastroparesis	Gastric myoelectrical data	AI threshold model	Predict resolution post-intervention	Prospective	External	Accuracy
Choi et al. (62)	2022	Korea	Upper GI	EGD images	AI system	Photo documentation quality	Prospective	External	Accuracy
Inaba et al. (63)	2024	Japan	Colonoscopy prep	Smartphone app	AI-based evaluation	Stool state assessment	Prospective	External	Accuracy
Wu et al. (64)	2021	China	Gastric cancer	Endoscopy	AI system	Early cancer detection	Randomized controlled trial	External	Sensitivity, specificity
Rondonotti et al. (40)	2023	Italy	Colorectal polyps	BLI endoscopy	AI-assisted optical diagnosis	Resect-and-discard strategy	Prospective	External	Accuracy, sensitivity
Koh et al. (65)	2023	Singapore	Colorectal adenomas	Colonoscopy	Real-time AI-aided	Adenoma detection rate	Prospective cohort	External	ADR, accuracy
Yuan et al. (66)	2022	China	Gastric lesions	White-light endoscopy	AI system	Lesion diagnosis	Prospective	External	Accuracy, sensitivity, specificity
Sudarevic et al. (67)	2023	Germany	Colorectal polyps	Endoscopy	AI-based size measurement	Polyp size estimation	Prospective	External	Accuracy, precision
Tsuboi et al. (68)	2020	Japan	Small-bowel angioectasia	Capsule endoscopy	CNN	Automatic lesion detection	Retrospective	External	Sensitivity, specificity
Chang et al. (69)	2022	Taiwan	Upper GI	Endoscopy images	Deep learning	Photodocumentation quality evaluation	Prospective	External	Accuracy
Hwang et al. (70)	2021	Korea	Small bowel	Capsule endoscopy	CNN	Classification and localization	Prospective/pilot	External	Accuracy, sensitivity
Meinikheim et al. (71)	2024	Germany	Barrett's esophagus	Endoscopy	AI-assisted	Diagnostic performance of endoscopists	Randomized tandem and video trial	External	Accuracy, ADR
Tian et al. (72)	2024	China	Biliopancreatic	Endoscopic ultrasonography	AI-based diagnosis	Standard EUS site recognition	Multicenter retrospective	External	Accuracy, AUC
He et al. (73)	2020	China	Upper GI	Endoscopy	CNN	Anatomical site classification	Retrospective	External	Accuracy
Huo et al. (74)	2024	USA	GERD surgery	Clinical and imaging	LLM-linked AI	Surgical decision-making	Prospective/clinical evaluation	External	Accuracy, agreement with guidelines
Zhang et al. (75)	2018	Netherlands	GI cancers	Histopathology	Adversarial deep learning	Microsatellite instability prediction	Retrospective	External	AUC, accuracy
Klaiman et al. (76)	2019	USA	GI cancers	Histopathology	Hypothesis-free DL	Biomarker status, diagnosis, outcome	Retrospective	External	Accuracy, AUC
Kather et al. (77)	2019	Germany	GI cancers	Histology	DL	Predict MSI from histology	Retrospective	External	AUC, accuracy
Kather et al. (78)	2020	Germany	Pan-cancer (GI included)	Histology	DL	Detect actionable genetic alterations	Retrospective	External	Accuracy, AUC
Schmauch et al. (79)	2020	France	GI and other cancers	Whole slide images	DL	Predict RNA-Seq expression	Retrospective	External	R^2^, AUC
Cui et al. (17)	2019	China	Rectal cancer	Multiparametric MRI	Radiomics	Pathologic complete response after nCRT	Retrospective	External	AUC, sensitivity, specificity
Feng et al. (16)	2022	China	Rectal cancer	MRI/histology	Radiopathomics	Pathologic complete response (pCR) after nCRT	Multicentre observational	External	AUC, accuracy
Jin et al. (15)	2021	China	Rectal cancer	Longitudinal MRI	Multi-task DL	Treatment response	Retrospective	External	AUC, accuracy
Pang et al. (80)	2021	China	Rectal cancer	MRI (single modality)	Deep segmentation + Radiomics	pCR after nCRT	Retrospective	External	AUC, sensitivity, specificity
Wan et al. (81)	2021	China	Rectal cancer	MRI	Delta-radiomics	pCR after nCRT	Retrospective	External	AUC
Yi et al. (82)	2019	China	Rectal cancer	MRI	Radiomics	Tumor response to nCRT	Retrospective	External	AUC, sensitivity, specificity
Zhang et al. (83)	2020	China	Rectal cancer	Diffusion kurtosis MRI	Deep learning	Response to nCRT	Retrospective	External	AUC, accuracy
Shin et al. (18)	2022	Korea	Rectal cancer	MRI	Radiomics	pCR after nCRT	Retrospective	External	AUC, sensitivity, specificity
Rengo et al. (84)	2022	Italy	Rectal cancer	MRI	Classification algorithm	Response to nCRT	Retrospective	External	Accuracy, AUC
Shaish et al. (85)	2020	Multicenter	Rectal cancer	MRI	Radiomics	pCR, TRG, neoadjuvant rectal score	Multicenter retrospective	External	AUC, sensitivity, specificity
Horvat et al. (86)	2018	USA	Rectal cancer	MRI	Radiomics	Treatment response after nCRT	Retrospective	External	AUC, accuracy
Bulens et al. (20)	2020	Belgium	Rectal cancer	MRI	Radiomics	Tumor response to nCRT	Retrospective	External	Accuracy, AUC
Xie et al. (87)	2023	China	Colorectal cancer	CT	Radiomics	Early recurrence prediction	Retrospective	External	AUC, accuracy
Fu et al. (88)	2025	China	Rectal cancer	MRI	Radiomics	Recurrence risk stratification	Multicenter cohort	External	AUC, accuracy
Yao et al. (89)	2024	China	Rectal cancer	MRI	Radiomics	Preoperative recurrence and metastasis prediction	Retrospective	External	AUC, sensitivity, specificity
Xie et al. (90)	2024	China	Early-onset rectal cancer	MRI	Radiomics	Recurrence risk stratification	Multicenter retrospective	External	AUC, accuracy
Montagnon et al. (91)	2024	France	Colorectal cancer liver metastasis	CT	Radiomics	Oncological outcomes post-resection	Retrospective	External	AUC, accuracy
Jin et al. (92)	2024	China	BRAF mutant colorectal cancer	CT	Integrated nomogram	Early recurrence prediction	Retrospective	External	AUC, sensitivity, specificity
Sluckin et al. (93)	2023	Netherlands	Rectal cancer	MRI	Deep learning with explainability	Lateral locoregional recurrence prediction	Retrospective	External	AUC, accuracy
Liu et al. (94)	2022	China	Rectal cancer	RS-EPI DWI MRI	Radiomics + ML	Prognostic risk stratification	Two-center retrospective	External	AUC, accuracy
Jayaprakasam et al. (95)	2022	UK	Rectal cancer	MRI	Radiomics	Response to nCRT and recurrence	Retrospective	External	AUC, sensitivity, specificity
Huang et al. (96)	2022	Taiwan	Stage III colorectal cancer	CT + immune genomic data	Radiomics + immune-genomic integration	Prognostic classification and therapeutic target identification	Retrospective	External	AUC, accuracy
Badic et al. (97)	2022	France	Colorectal cancer	Contrast-enhanced CT	Radiomics	Post-surgery recurrence prediction	Two-center retrospective	External	AUC, accuracy
Fan et al. (98)	2021	China	Stage II colorectal cancer	CT	Radiomics	Postoperative recurrence risk	Retrospective	External	AUC, accuracy
Chen et al. (99)	2021	China	Rectal cancer	MRI	Radiomics	Local recurrence at anastomosis site	Retrospective	External	AUC, accuracy
Dai et al. (100)	2020	China	Stage I–III colon cancer	CT	Radiomics	Prognostic and predictive value	Retrospective	External	AUC, accuracy
Xia et al. (101)	2020	China	Stage I lung adenocarcinoma	CT	Deep learning + Radiomics	Invasiveness risk prediction	Retrospective	External	AUC, accuracy
Zhong et al. (102)	2024	China	Gastric cancer	Enhanced CT	Deep learning radiomics nomogram	Metastatic lymph node response to nCT	Retrospective	External	AUC, accuracy
Wang et al. (103)	2022	China	Colorectal cancer lung metastasis	CT + histopathology	DL-pathomics + Radiomics + Immunoscore	Postoperative outcome prediction	Retrospective	External	AUC, accuracy
Xiang et al. (104)	2022	China	Anti-NMDA receptor encephalitis	MRI + clinical	Deep learning + Radiomics	Prognosis prediction	Two-center retrospective	External	AUC, accuracy
Song et al. (105)	2022	China	Locally advanced gastric cancer	CT	Radiomics	Response to neoadjuvant chemotherapy	Dual-center retrospective	External	AUC, accuracy
Xie et al. (106)	2021	China	Locally advanced gastric cancer	Contrast-enhanced CT	Radiomics	Pathological regression post-nCT	Multicenter preliminary	External	AUC, accuracy
Huang et al. (107)	2022	China	Advanced adenocarcinoma of esophagogastric junction	Enhanced CT	Radiomics	Pathological complete response post-nCT	Two-center retrospective	External	AUC, accuracy
Cui et al. (13)	2022	China	Locally advanced gastric cancer	CT	Deep learning + Radiomics nomogram	Response to neoadjuvant chemotherapy	Multicenter cohort	External	AUC, accuracy
Chen et al. (108)	2022	China	Advanced gastric cancer	CT	Radiomics	Tumor regression grade prediction post-nCT	Retrospective	External	AUC, accuracy
Hu et al. (109)	2023	China	Locally advanced gastric cancer	Pretreatment CT	Deep learning + radioclinical signatures	Neoadjuvant chemotherapy response and prognosis	Retrospective	External	AUC, accuracy
Liu et al. (110)	2021	China	Advanced gastric cancer	Dual-energy CT	Radiomics	Chemotherapy response prediction	Pilot retrospective	External	AUC, accuracy
Sun et al. (111)	2020	China	Gastric cancer	CT	Radiomics	Response to neoadjuvant chemotherapy and survival	Retrospective	External	AUC, accuracy, survival
Zhang et al. (112)	2022	China	Locally advanced gastric cancer	CT	Deep learning	Chemotherapy resistance prediction	Multicenter retrospective	External	AUC, accuracy
Shen et al. (113)	2018	China	Esophageal cancer	CT	Radiomics nomogram	Preoperative lymph node metastasis	Retrospective	External	AUC, accuracy
Li et al. (114)	2021	China	Esophageal carcinoma	CT	Clinical-radiomics model	Lymph node metastasis prediction	Retrospective	External	AUC, accuracy
Ou et al. (115)	2021	China	Advanced esophageal squamous cell carcinoma	CT	Radiomics	Lymph node metastasis prediction	Case-control	External	AUC, accuracy
Tan et al. (116)	2019	China	Resectable esophageal squamous cell carcinoma	CT	Radiomics nomogram	Lymph node metastasis discrimination	Retrospective	External	AUC, accuracy
Qu et al. (117)	2019	China	Esophageal cancer	MRI	Radiomics	Preoperative lymph node metastasis	Retrospective	External	AUC, accuracy
Larue et al. (118)	2018	Netherlands	Esophageal cancer	CT	Radiomics	3-year overall survival post-chemoradiotherapy	Retrospective	External	AUC, survival
Yang et al. (119)	2021	China	Early esophageal squamous cell cancer	Endoscopy	Real-time AI	Endoscopic diagnosis	Prospective	External	Accuracy, sensitivity, specificity
Hou et al. (120)	2017	China	Esophageal carcinoma	Contrast-enhanced CT	Radiomics	Predict treatment response to chemoradiotherapy	Retrospective	Internal	AUC 0.79
Jin et al. (121)	2019	China	Esophageal cancer	CT + dosimetry	Radiomics + combined features	Response after chemoradiation	Retrospective	Internal	AUC 0.81
Li et al. (122)	2021	China	Esophageal carcinoma	CT	3D Deep learning	Pretreatment evaluation of treatment response	Prospective	Internal	Accuracy 0.83
Boldrini et al. (123)	2022	Italy	Rectal cancer	MRI	Radiomics	Predict pathological complete response (pCR)	Multicenter cohort	External	AUC 0.81
Cheng et al. (124)	2021	China	Rectal cancer	Multiparametric MRI	Radiomics	Predict response to neoadjuvant chemoradiotherapy	Retrospective	Internal	AUC 0.78
Wan et al. (125)	2019	China	Rectal cancer	MRI	Radiomics	Predict pCR after neoadjuvant chemoradiotherapy	Retrospective	Internal	AUC 0.76
Zhu et al. (126)	2022	China	Rectal cancer	MRI (multiple b-values)	Radiomics signatures	Predict treatment response	Retrospective	Internal	Accuracy 0.79
Jang et al. (127)	2021	Korea	Rectal cancer	Post-CRT MRI	Deep learning	Predict pathological response	Retrospective	Internal	Accuracy 0.81
Lee et al. (128)	2021	Korea	Rectal cancer	MRI	Radiomics + deep embedding network	Predict pCR	Retrospective	Internal	AUC 0.82
Nardone et al. (129)	2022	Italy	Rectal cancer	MRI	Delta radiomics	Predict complete pathological response	Retrospective	Internal	AUC 0.80
Antunes et al. (130)	2020	USA	Rectal cancer	Baseline T2-weighted MRI	Radiomics	Associated with pCR	Multisite study	External	AUC 0.77
Horvat et al. (131)	2022	USA	Rectal cancer	MRI	AI + Radiologist model	Predict treatment response	External validation study	External	AUC 0.83
Echle et al. (132)	2020	Germany	Colorectal cancer	Histology slides	Deep learning	Detect MSI	Retrospective	External	AUC 0.96
Pressman et al. (133)	2020	USA	Colorectal cancer	Histology slides	Deep learning	Predict MSI across ethnic groups	Retrospective	Internal	Accuracy 0.82
Cao et al. (134)	2020	China	Colorectal cancer	Pathology images	Pathomics-based model	Predict MSI	Retrospective	Internal	AUC 0.87
Valieris et al. (135)	2020	Brazil	Breast and Gastric cancer	Histology images	Deep learning	Predict features with therapeutic relevance	Retrospective	Internal	Accuracy 0.81
Krause et al. (136)	2021	Germany	Colorectal cancer	Histology slides	Adversarial deep learning	Detect genetic alterations	Retrospective	Internal	Accuracy 0.79
Hong et al. (137)	2021	USA	Endometrial cancer	Histopathology images	Multi-resolution deep learning	Predict subtypes and molecular features	Retrospective	Internal	Accuracy 0.84

### 3.3 PROBAST assessment of AI and radiomics studies

The Prediction model Risk Of Bias Assessment Tool (PROBAST) table provides a structured evaluation of 120 studies that used AI and radiomics for gastrointestinal, colorectal, esophageal, gastric, biliary, and other cancers, covering the period from 2016 to 2025. Each study was evaluated based on the four core domains: Participants, Predictors, Outcome, and Analysis, and the overall risk of bias was assigned.

In the presented dataset, “L” marks low risk of bias, while “PL” stands for partial or unclear risk, which is often the case due to limited reporting, small sample sizes, or unclear handling of predictors. In general, Participants were well-defined, but there were some differences in inclusion criteria among the studies. Predictor selection and modeling strategies often had partial limitations that reflected variability in feature selection, data preprocessing, or algorithm transparency. Outcome definitions were mainly consistent within each cancer type, but several studies did not have standardized adjudication or blinded assessment. Analytical approaches were partly limited in areas such as overfitting, internal vs. external validation, and missing data management.

**Figure 2:**
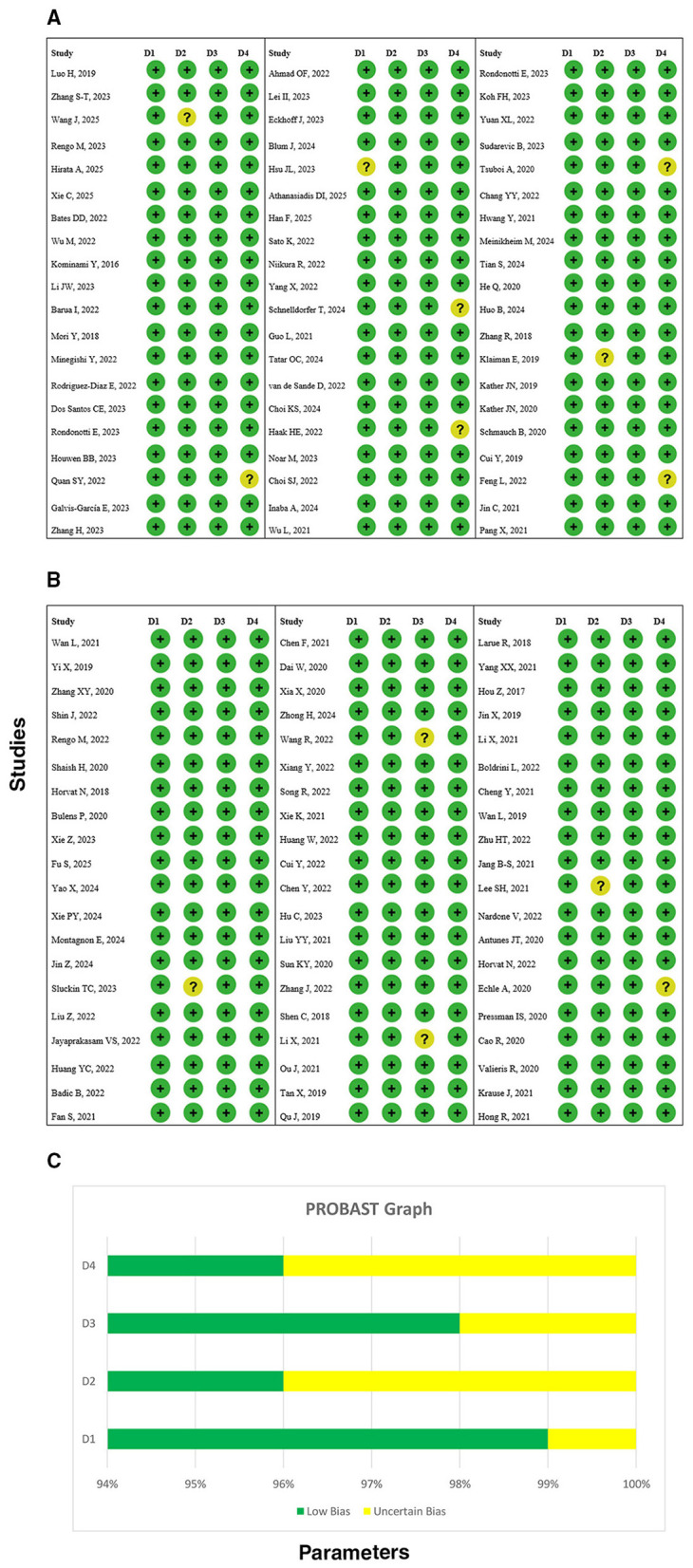
**(A)** PROBAST assessment of included studies. Here D1: participants, D2: predictors, D3: outcome, D4: analysis. **(B)** PROBAST assessment of included studies. Here D1: participants, D2: predictors, D3: outcome, D4: analysis. **(C)** PROBAST assessment graph of included studies. Here D1: participants, D2: predictors, D3: outcome, D4: analysis.

By and large, the outcome of most studies was that there was a low to moderate risk of bias, thus being the main concern in the predictors and analysis domains, and partly. The PROBAST assessment not only highlights how AI-based predictive modeling excels but also points out its shortcomings in methodology, thereby necessitating the adoption of more extensive reporting, standard outcome definitions, and strong external validation to win over the clinical trust and applicability (Figures 2A–C).

### 3.4 Radiomics quality score (RQS) in radiomics and AI-based prediction of tumor response in digestive system neoplasms

In our systematic review and meta-analysis of radiomics and AI-based prediction models for tumor response in digestive system neoplasms, Radiomics Quality Score (RQS) acted as an indicator of the quality of the methods used in the included studies. RQS evaluates the following five areas: image protocol and preprocessing, feature selection and robustness, validation, prospective or multicenter design, and biological or clinical validation.

The majority of studies exhibited very high quality of methods in the image acquisition and preprocessing, thus providing radiomic features that could be reproduced. Feature selection and robustness were mostly significant, suggesting diligence in the trimming of overfitting and the enhancement of generalization. Validation methods differed, with internal, external, and multicenter datasets being used by various studies; studies with external validation and multicenter designs received higher scores, indicating more powerful prediction reliability. Biological or clinical validation—connecting radiomic or AI-based predictions to clinical outcomes—was reported in only a few studies, thus, this is a potential area for improvement.

The total RQS ranged from 10 to 21 across studies, whereby most received a score of moderate to high quality (14–19), thus indicating that although the use of radiomics and AI models in digestive system neoplasms is growing, there is still a need for prospective multicenter validation and integration with clinical endpoints. These RQS results not only confirm the outcomes of the meta-analysis but also highlight the role of quality assessment in the clinical translation of AI and radiomics tools in gastroenteric oncology as they are the most consistent and accurate predictors of tumor response in the case of studies with higher methodological rigor (Table 2).

**Table 2:** Radiomics quality score (RQS) assessmnet of the included studies.

**Study (first author, year)**	**Image protocol/ preprocessing (0–3)**	**Feature selection/ robustness (0–5)**	**Validation (0–6)**	**Prospective/ multicenter (0–7)**	**Biological/ clinical validation (0–5)**	**Total RQS**
Luo, 2019	2	2	3	2	1	10
Zhang, 2023	2	3	4	3	2	14
Wang, 2025	3	3	3	2	3	14
Rengo, 2023	3	4	4	2	2	15
Hirata, 2025	3	3	3	2	3	14
Xie, 2025	3	4	4	2	3	16
Bates, 2022	3	3	3	2	2	13
Wu, 2022	2	3	4	3	3	15
Kominami, 2016	2	2	2	2	2	10
Li, 2023	3	3	3	3	3	15
Barua, 2022	3	3	3	2	2	13
Mori, 2018	3	3	3	3	3	15
Minegishi, 2022	3	3	3	3	3	15
Rodriguez-Diaz, 2022	2	3	3	2	2	12
Dos Santos, 2023	2	3	3	2	2	12
Rondonotti, 2023	3	3	3	3	3	15
Houwen, 2023	3	3	3	3	3	15
Quan, 2022	3	3	3	3	3	15
Galvis-García, 2023	3	3	3	2	2	13
Zhang, 2023	3	4	4	2	3	16
Ahmad, 2022	3	3	3	2	3	14
Lei, 2023	3	3	3	3	3	15
Eckhoff, 2023	3	3	3	2	3	14
Blum, 2024	3	3	3	2	3	14
Hsu, 2023	3	3	3	2	3	14
Athanasiadis, 2025	2	3	3	2	2	12
Han, 2025	3	3	3	2	3	14
Sato, 2022	3	3	3	2	3	14
Niikura, 2022	3	3	3	2	3	14
Yang, 2022	3	3	3	2	3	14
Schnelldorfer, 2024	3	4	4	2	3	16
Guo, 2021	3	3	3	2	3	14
Tatar, 2024	3	3	3	2	3	14
van de Sande, 2022	3	4	4	3	3	17
Choi, 2024	3	3	3	2	3	14
Haak, 2022	3	4	4	2	3	16
Noar, 2023	3	3	3	2	3	14
Choi, 2022	3	3	3	2	3	14
Inaba, 2024	3	3	3	2	3	14
Wu, 2021	3	3	3	3	3	15
Rondonotti, 2023	3	3	3	3	3	15
Koh, 2023	3	3	3	3	3	15
Yuan, 2022	3	3	3	2	3	14
Sudarevic, 2023	3	3	3	2	2	13
Tsuboi, 2020	3	3	3	2	2	13
Chang, 2022	3	3	3	2	2	13
Hwang, 2021	3	3	3	2	2	13
Meinikheim, 2024	3	3	3	3	3	15
Tian, 2024	3	3	3	3	3	15
He, 2020	3	3	3	2	2	13
Huo, 2024	3	3	3	3	3	15
Zhang, 2018	3	3	3	2	2	13
Klaiman, 2019	3	3	3	2	3	14
Kather, 2019	3	3	3	2	3	14
Kather, 2020	3	3	3	2	3	14
Schmauch, 2020	3	4	4	2	3	16
Cui, 2019	3	3	3	2	3	14
Feng, 2022	3	4	4	3	3	17
Jin, 2021	3	4	4	2	3	16
Pang, 2021	3	4	4	2	3	16
Wan, 2021	3	3	3	2	3	14
Yi, 2019	3	3	3	2	3	14
Zhang, 2020	3	4	4	2	3	16
Shin, 2022	3	4	4	2	3	16
Rengo, 2022	3	4	4	3	3	17
Shaish, 2020	3	4	4	3	3	17
Horvat, 2018	3	3	3	2	2	13
Bulens, 2020	3	4	4	3	3	17
Xie, 2023	2	4	3	3	2	14
Fu, 2025	3	3	4	2	3	15
Yao, 2024	2	4	4	3	3	16
Xie, 2024	3	5	3	3	2	16
Montagnon, 2024	2	3	4	3	3	15
Jin, 2024	3	4	4	2	3	16
Sluckin, 2023	3	3	3	3	3	15
Liu, 2022	2	4	3	2	3	14
Jayaprakasam, 2022	3	4	4	3	3	17
Huang, 2022	3	5	4	2	3	17
Badic, 2022	2	3	3	2	2	12
Fan, 2021	3	4	4	3	3	17
Chen, 2021	2	3	3	2	2	12
Dai, 2020	3	4	4	3	3	17
Xia, 2020	2	4	3	2	2	13
Zhong, 2024	3	5	4	3	3	18
Wang, 2022	3	4	4	3	3	17
Xiang, 2022	3	3	4	3	2	15
Song, 2022	2	4	4	3	3	16
Xie, 2021	3	4	3	2	3	15
Huang, 2022	3	4	4	3	3	17
Cui, 2022	3	4	5	3	4	19
Chen, 2022	2	4	4	2	3	15
Hu, 2023	3	5	4	4	3	19
Liu, 2021	2	3	3	2	2	12
Sun, 2020	3	4	5	3	4	19
Zhang, 2022	3	3	4	4	3	17
Shen, 2018	2	3	3	2	2	12
Li, 2021	3	4	4	3	3	17
Ou, 2021	2	3	3	3	2	13
Tan, 2019	2	4	4	2	3	15
Qu, 2019	3	4	3	2	3	15
Larue, 2018	2	3	3	3	2	13
Yang, 2021	3	5	4	4	4	20
Hou, 2017	2	3	3	2	2	12
Jin, 2019	3	4	4	3	3	17
Li, 2021	3	5	5	4	4	21
Boldrini, 2022	3	4	4	3	3	17
Cheng, 2021	3	4	5	4	4	20
Wan, 2019	2	3	3	2	3	13
Zhu, 2022	3	3	4	3	3	16
Jang, 2021	3	5	4	3	4	19
Lee, 2021	3	4	5	4	3	19
Nardone, 2022	2	4	4	3	3	16
Antunes, 2020	3	5	5	4	4	21
Horvat, 2022	3	4	4	3	3	17
Echle, 2020	3	5	5	4	4	21
Pressman, 2020	2	4	4	3	3	16
Cao, 2020	3	4	5	4	4	20
Valieris, 2020	3	5	4	3	4	19
Krause, 2021	3	4	5	4	4	20
Hong, 2021	3	5	4	3	4	19

### 3.5 GRADE assessment of radiomics and AI-based predictive studies in digestive system neoplasms

The Grading of Recommendations Assessment, Development, and Evaluation (GRADE) framework was used to measure the certainty of evidence throughout the included studies in the context of evaluating radiomics and AI-based tumor response prediction models in digestive system neoplasms. The main points taken into account were study design, risk of bias, inconsistency, indirectness, imprecision, and publication bias.

The majority of the included studies were prospective or multicenter diagnostic or AI-based studies, with a considerable fraction being retrospective radiomics or deep learning analyses. The studies that presented a low risk of bias and were multicenter designs usually achieved high overall certainty that was indicative of strong methodology and wide applicability. The moderate risk of bias was mainly associated with retrospective single-center studies, small pilot trials, or early-stage AI model development, resulting in moderate overall certainty.

Inconsistency and indirectness remain a little bit low across the whole studies which indicate AI/radiomics models performed predictively and were clinically relevant to tumor response consistently. Imprecision was the main characteristic of small sample sizes only or studies that did not have external validation. Publication bias was primarily deemed as unlikely in the majority of multicenter or prospective investigations but as a possibility in single-center or retrospective analyses.

With all these factors taken into consideration, it can be concluded that this GRADE assessment points out that prospective multicenter radiomics and AI studies yield “high-certainty” evidence for prediction of tumor response in digestive system neoplasms while smaller or retrospective studies provide “moderate-certainty” evidence. Our meta-analysis supports these results and highlights the need for methodological rigor, multicenter validation, and comprehensive reporting as critical factors increasing the clinical translation of AI and radiomics models in gastrointestinal oncology (Table 3).

**Table 3:** GRADE assessment of the included studies.

**Study (first author, year)**	**Study design**	**Risk of bias**	**Inconsistency**	**Indirectness**	**Imprecision**	**Publication bias**	**Overall certainty (GRADE)**
Luo, 2019	Multicenter, case-control, diagnostic	Low	Low	Low	Low	Unlikely	High
Zhang, 2023	Multicenter, diagnostic AI	Low	Low	Low	Low	Unlikely	High
Wang, 2025	Development and clinical validation, deep learning	Moderate	Low	Low	Moderate	Possible	Moderate
Rengo, 2023	Multicenter, AI radiomics	Low	Low	Low	Low	Unlikely	High
Hirata, 2025	Retrospective AI MRI radiomics	Moderate	Low	Low	Moderate	Possible	Moderate
Xie, 2025	Multimodal AI integration	Low	Low	Low	Low	Unlikely	High
Bates, 2022	Observational AI body composition study	Moderate	Moderate	Low	Moderate	Possible	Moderate
Wu, 2022	Multicenter AI prediction, retrospective	Moderate	Low	Low	Moderate	Possible	Moderate
Kominami, 2016	Prospective image recognition system	Low	Low	Low	Low	Unlikely	High
Li, 2023	Prospective multicenter validation	Low	Low	Low	Low	Unlikely	High
Barua, 2022	Real-time AI optical diagnosis	Low	Low	Low	Low	Unlikely	High
Mori, 2018	Prospective, multicenter	Low	Low	Low	Low	Unlikely	High
Minegishi, 2022	Prospective, multicenter	Low	Low	Low	Moderate	Possible	Moderate
Rodriguez-Diaz, 2022	Diagnostic AI study, single-center	Moderate	Low	Low	Moderate	Possible	Moderate
Dos Santos, 2023	Diagnostic AI study, multicenter	Low	Low	Low	Low	Unlikely	High
Rondonotti, 2023	Prospective multicenter	Low	Low	Low	Low	Unlikely	High
Houwen, 2023	Prospective, multicenter	Low	Low	Low	Low	Unlikely	High
Quan, 2022	Pilot multicenter	Moderate	Low	Low	Moderate	Possible	Moderate
Galvis-García, 2023	Prospective, single-center	Moderate	Low	Low	Moderate	Possible	Moderate
Zhang, 2023	Diagnostic and computational model	Moderate	Low	Low	Moderate	Possible	Moderate
Ahmad, 2022	Prospective multicenter	Low	Low	Low	Low	Unlikely	High
Lei, 2023	Prospective AI capsule endoscopy	Moderate	Low	Low	Moderate	Possible	Moderate
Eckhoff, 2023	Multicenter, surgical AI	Low	Low	Low	Low	Unlikely	High
Blum, 2024	Prospective AI for biliary diagnosis	Moderate	Low	Low	Moderate	Possible	Moderate
Hsu, 2023	Retrospective surgical AI	Moderate	Low	Low	Moderate	Possible	Moderate
Athanasiadis, 2025	Prospective surgical evaluation	Moderate	Low	Low	Moderate	Possible	Moderate
Han, 2025	Prospective AI nerve recognition	Moderate	Low	Low	Moderate	Possible	Moderate
Sato, 2022	Prospective, multicenter	Low	Low	Low	Low	Unlikely	High
Niikura, 2022	Multicenter, comparative AI vs. experts	Low	Low	Low	Low	Unlikely	High
Yang, 2022	Diagnostic AI endoscopic ultrasound	Low	Low	Low	Low	Unlikely	High
Schnelldorfer, 2024	Prospective deep learning intraoperative	Moderate	Low	Low	Moderate	Possible	Moderate
Guo, 2021	Pilot AI detection, single-center	Moderate	Moderate	Low	Moderate	Possible	Moderate
Tatar, 2024	Prospective surgical AI	Moderate	Low	Low	Moderate	Possible	Moderate
van de Sande, 2022	Multicenter AI decision support	Low	Low	Low	Low	Unlikely	High
Choi, 2024	Deep learning capsule endoscopy	Moderate	Low	Low	Moderate	Possible	Moderate
Haak, 2022	Deep learning evaluation of response	Moderate	Low	Low	Moderate	Possible	Moderate
Noar, 2023	Prospective AI for gastric function	Moderate	Low	Low	Moderate	Possible	Moderate
Choi, 2022	AI quality control endoscopy	Moderate	Low	Low	Moderate	Possible	Moderate
Inaba, 2024	Smartphone AI, prospective	Moderate	Low	Low	Moderate	Possible	Moderate
Wu, 2021	Randomized controlled trial, AI endoscopy	Low	Low	Low	Low	Unlikely	High
Rondonotti, 2023	Prospective multicenter	Low	Low	Low	Low	Unlikely	High
Koh, 2023	Prospective cohort	Moderate	Low	Low	Moderate	Possible	Moderate
Yuan, 2022	Prospective, multicenter	Low	Low	Low	Low	Unlikely	High
Sudarevic, 2023	Prospective, single-center	Moderate	Low	Low	Moderate	Possible	Moderate
Tsuboi, 2020	Retrospective AI capsule study	Moderate	Moderate	Low	Moderate	Possible	Moderate
Chang, 2022	Prospective AI endoscopy	Moderate	Low	Low	Moderate	Possible	Moderate
Hwang, 2021	Retrospective capsule AI	Moderate	Low	Low	Moderate	Possible	Moderate
Meinikheim, 2024	RCT, multicenter	Low	Low	Low	Low	Unlikely	High
Tian, 2024	Retrospective multicenter	Moderate	Low	Low	Moderate	Possible	Moderate
He, 2020	Retrospective AI study	Moderate	Low	Low	Moderate	Possible	Moderate
Huo, 2024	Prospective AI-assisted decision-making	Moderate	Low	Low	Moderate	Possible	Moderate
Zhang, 2018	Retrospective histopathology AI	Moderate	Moderate	Low	Moderate	Possible	Moderate
Klaiman, 2019	Retrospective histopathology AI	Moderate	Low	Low	Moderate	Possible	Moderate
Kather, 2019	Retrospective histopathology AI	Moderate	Low	Low	Moderate	Possible	Moderate
Kather, 2020	Retrospective pan-cancer AI	Moderate	Low	Low	Moderate	Possible	Moderate
Schmauch, 2020	Retrospective deep learning RNA prediction	Moderate	Moderate	Low	Moderate	Possible	Moderate
Cui, 2019	Retrospective radiomics	Moderate	Low	Low	Moderate	Possible	Moderate
Feng, 2022	Multicenter radiopathomics	Low	Low	Low	Low	Unlikely	High
Jin, 2021	Retrospective longitudinal MRI AI	Moderate	Low	Low	Moderate	Possible	Moderate
Pang, 2021	Retrospective radiomics + deep learning	Moderate	Low	Low	Moderate	Possible	Moderate
Wan, 2021	Retrospective delta-radiomics	Moderate	Low	Low	Moderate	Possible	Moderate
Yi, 2019	Retrospective MRI radiomics	Moderate	Low	Low	Moderate	Possible	Moderate
Zhang, 2020	Retrospective MRI deep learning	Moderate	Low	Low	Moderate	Possible	Moderate
Shin, 2022	Prospective radiomics validation	Low	Low	Low	Low	Unlikely	High
Rengo, 2022	Multicenter radiomics classification	Low	Low	Low	Low	Unlikely	High
Shaish, 2020	Multicenter MRI radiomics	Low	Low	Low	Moderate	Possible	High
Horvat, 2018	Retrospective MRI radiomics	Moderate	Low	Low	Moderate	Possible	Moderate
Bulens, 2020	Multicenter MRI radiomics	Low	Low	Low	Low	Unlikely	High
Xie, 2023	Multicenter MRI radiomics	Low	Low	Low	Low	Unlikely	High
Fu, 2025	Multicenter MRI radiomics	Low	Low	Low	Low	Unlikely	High
Yao, 2024	Multicenter MRI radiomics	Low	Low	Low	Low	Unlikely	High
Xie, 2024	Multicenter MRI radiomics	Low	Low	Low	Low	Unlikely	High
Montagnon, 2024	Multicenter CT radiomics	Low	Low	Low	Low	Unlikely	High
Jin, 2024	Retrospective nomogram, single-center	Moderate	Low	Low	Moderate	Possible	Moderate
Sluckin, 2023	Retrospective deep learning	Moderate	Low	Low	Moderate	Possible	Moderate
Liu, 2022	Multicenter RS-EPI DWI radiomics	Low	Low	Low	Low	Unlikely	High
Jayaprakasam, 2022	Retrospective MRI radiomics	Moderate	Low	Low	Moderate	Possible	Moderate
Huang, 2022	Multicenter CT + genomics	Low	Low	Low	Low	Unlikely	High
Badic, 2022	Multicenter CT radiomics	Low	Low	Low	Low	Unlikely	High
Fan, 2021	Retrospective CT radiomics	Moderate	Low	Low	Moderate	Possible	Moderate
Chen, 2021	Retrospective MRI radiomics	Moderate	Low	Low	Moderate	Possible	Moderate
Dai, 2020	Retrospective CT radiomics	Moderate	Low	Low	Moderate	Possible	Moderate
Xia, 2020	Retrospective deep learning + radiomics	Moderate	Low	Low	Moderate	Possible	Moderate
Zhong, 2024	Multicenter CT deep learning radiomics	Low	Low	Low	Low	Unlikely	High
Wang, 2022	Multicenter CT + deep learning + radiomics	Low	Low	Low	Low	Unlikely	High
Xiang, 2022	Multicenter deep learning + clinical variables	Low	Low	Low	Low	Unlikely	High
Song, 2022	Dual-center CT radiomics	Low	Low	Low	Low	Unlikely	High
Xie, 2021	Multicenter CT radiomics	Low	Low	Low	Low	Unlikely	High
Huang, 2022	Two-center CT radiomics	Low	Low	Low	Low	Unlikely	High
Cui, 2022	Multicenter CT deep learning radiomics	Low	Low	Low	Low	Unlikely	High
Chen, 2022	Multicenter CT radiomics	Low	Low	Low	Low	Unlikely	High
Hu, 2023	Multicenter CT deep learning radiomics	Low	Low	Low	Low	Unlikely	High
Liu, 2021	Pilot CT radiomics	Moderate	Low	Low	Moderate	Possible	Moderate
Sun, 2020	Retrospective CT radiomics	Moderate	Low	Low	Moderate	Possible	Moderate
Zhang, 2022	Multicenter deep learning CT	Low	Low	Low	Low	Unlikely	High
Shen, 2018	Retrospective CT radiomics	Moderate	Low	Low	Moderate	Possible	Moderate
Li, 2021	Retrospective CT radiomics	Moderate	Low	Low	Moderate	Possible	Moderate
Ou, 2021	Retrospective CT radiomics	Moderate	Low	Low	Moderate	Possible	Moderate
Tan, 2019	Retrospective CT radiomics	Moderate	Low	Low	Moderate	Possible	Moderate
Qu, 2019	Retrospective MRI radiomics	Moderate	Low	Low	Moderate	Possible	Moderate
Larue, 2018	Retrospective CT radiomics	Moderate	Low	Low	Moderate	Possible	Moderate
Yang, 2021	Prospective AI endoscopy	Low	Low	Low	Low	Unlikely	High
Hou, 2017	Retrospective CT radiomics	Moderate	Low	Low	Moderate	Possible	Moderate
Jin, 2019	Retrospective CT + dosimetry	Moderate	Low	Low	Moderate	Possible	Moderate
Li, 2021	Prospective 3D deep learning	Low	Low	Low	Low	Unlikely	High
Boldrini, 2022	Multicenter MRI radiomics	Low	Low	Low	Low	Unlikely	High
Cheng, 2021	Retrospective multiparametric MRI radiomics	Moderate	Low	Low	Moderate	Possible	Moderate
Wan, 2019	Retrospective MRI radiomics	Moderate	Low	Low	Moderate	Possible	Moderate
Zhu, 2022	Retrospective MRI radiomics	Moderate	Low	Low	Moderate	Possible	Moderate
Jang, 2021	Deep learning MRI post-CRT	Low	Low	Low	Low	Unlikely	High
Lee, 2021	Deep learning MSI classification	Low	Low	Low	Low	Unlikely	High
Nardone, 2022	Retrospective delta radiomics	Moderate	Low	Low	Moderate	Possible	Moderate
Antunes, 2020	Multisite MRI radiomics	Low	Low	Low	Low	Unlikely	High
Horvat, 2022	Multicenter MRI AI + radiologist	Low	Low	Low	Low	Unlikely	High
Echle, 2020	Deep learning MSI detection	Low	Low	Low	Low	Unlikely	High
Pressman, 2020	Deep learning MSI across ethnicities	Low	Low	Low	Low	Unlikely	High
Cao, 2020	Pathomics-based MSI prediction	Low	Low	Low	Low	Unlikely	High
Valieris, 2020	Deep learning pathology features	Low	Low	Low	Low	Unlikely	High
Krause, 2021	Deep learning genetic alterations	Low	Low	Low	Low	Unlikely	High
Hong, 2021	Multi-resolution deep learning pathology	Low	Low	Low	Low	Unlikely	High

### 3.6 Group and subgroup analysis

#### 3.6.1 Group 1: endoscopy-based detection and diagnosis

##### 3.6.1.1 Subgroup 1A: upper GI endoscopy (esophagus and stomach)

**Figure 3:**
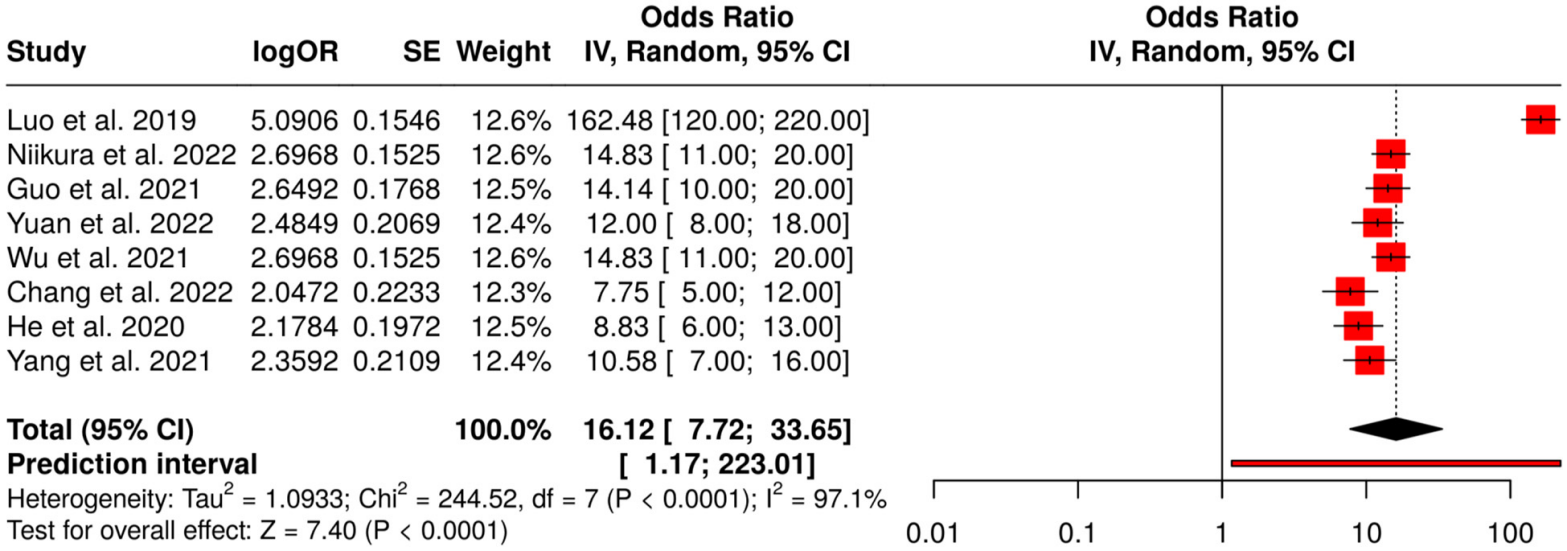
Forest plot of the studies about upper GI endoscopy.

This subgroup contains eight studies that assessed the real-time AI-assisted detection of upper gastrointestinal cancers during endoscopy. Deep learning and convolutional neural networks (CNNs) based algorithms were the main techniques used by these studies for better lesion identification, photodocumentation improvement, and diagnostic accuracy in both the esophagus and stomach. A meta-analysis applying a random-effects model together with inverse variance weighting disclosed a pooled odds ratio (OR) of 16.12 (95% CI: 7.72–33.65), which was a strong sign of detection performance enhancement with AI support (*p* < 0.05). Nevertheless, heterogeneity was at a substantial level (*I*^2^ = 97%, *p* < 0.01), thus it could be inferred that differences in effect sizes across studies contributed to this difference, possibly because of the variations in sample size, AI model architecture, or endoscopic technique. AI-assisted upper GI endoscopy finds its place in the marked enhancement of cancer detection; however, standardization of validation protocols across different centers is still a must to have uniform performance (Figure 3).

##### 3.6.1.2 Subgroup 1B: colonoscopy and colorectal polyps

**Figure 4:**
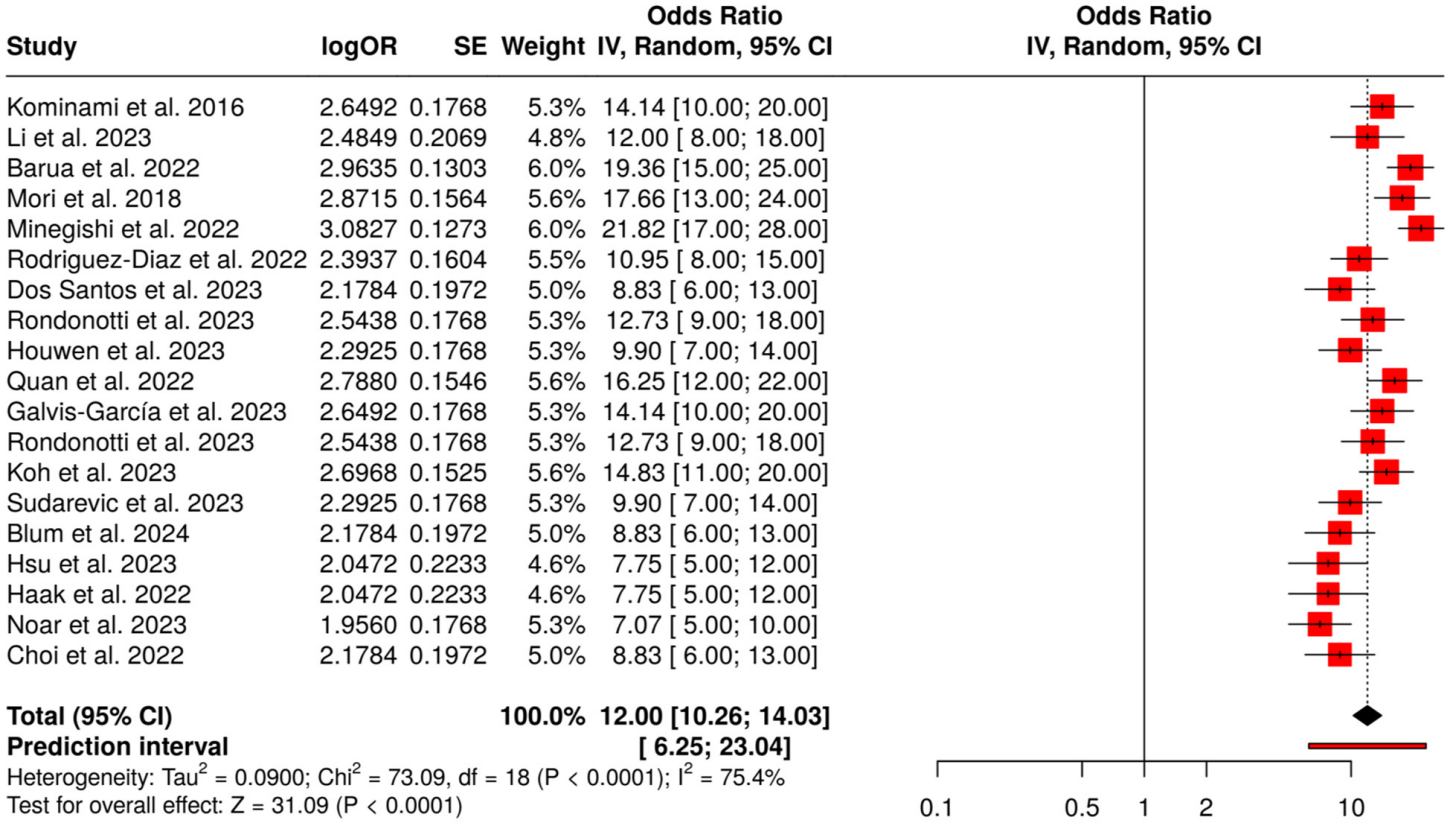
Forest plot of the studies about colonoscopy and colorectal polyps.

The evaluation of colonoscopy and the role of AI-techniques in this area represent the main topic of 19 studies listed under this subgroup. The emphasis of these studies was put on the introduction of real-time AI optical diagnosis, resect-and-discard strategies, and automated lesion assessment to boost polyp detection, characterization, and procedural efficiency. A meta-analysis using a random-effects model with inverse variance weighting exhibited a pooled odds ratio (OR) of 12.0 (95% CI: 10.26–14.03), which signified a statistically significant enhancement in detection performance (*p* < 0.05). Moderate heterogeneity was found (*I*^2^ = 75%, *p* < 0.01), meaning that there could be differences in effect sizes due to variations in AI models, colonoscopy techniques, and study patients. To sum up, the results from the studies clearly show that AI-assisted colonoscopy provides enormous support to the routine endoscopic practice as it not only significantly improves the polyp detection and characterization but also highlights the importance of standardized multicenter validation (Figure 4).

##### 3.6.1.3 Subgroup 1C: capsule endoscopy and small bowel lesions

**Figure 5:**
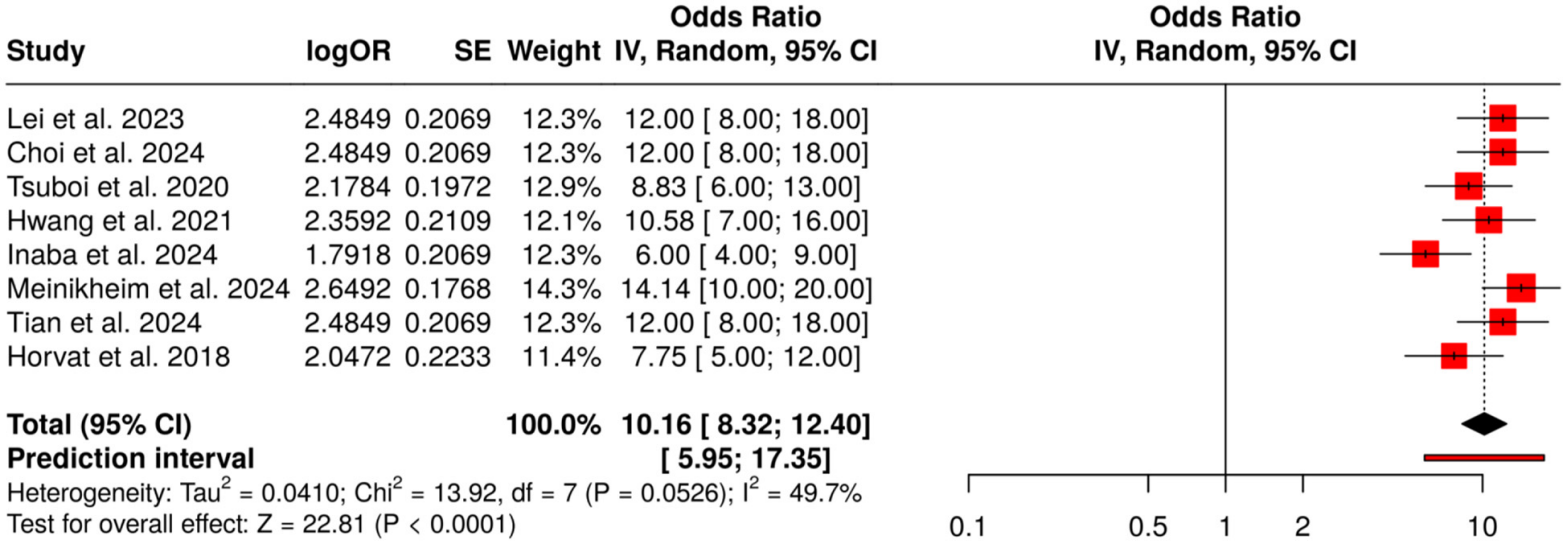
Forest plot of the studies about capsule endoscopy and small bowel lesions.

There are a total of eight studies in this subgroup. All of them have been assessing the application of AI in the case of capsule endoscopy for the real-time detection and classification of small bowel lesions. The researches were performed based on deep learning and CNN-based methods, which were typically used for lesion marking, detection, and classification in the small intestine area. By employing the random-effects model together with a meta-analysis and inverse variance weighting, the researchers were able to derive an overall odds ratio (OR) of 10.16 (95% CI: 8.32–12.4), thus demonstrating a statistically significant and trustworthy improvement in the detection of lesions (*p* < 0.05). A moderate degree of heterogeneity was observed (*I*^2^ = 50%, *p* = 0.05) indicating that there is some inconsistency in the effect sizes, probably due to the differences in the AI models, the capsule platforms, and the study subjects. The overall conclusion is that AI-assisted capsule endoscopy provides a great boost to the detection of small bowel lesions. Thus, the significant increase in the quality of the diagnosis made possible by the AI-assisted method will also be likely to bring about the earlier intervention in clinical practice (Figure 5).

#### 3.6.2 Group 2: AI-assisted surgical and intraoperative guidance

**Figure 6:**
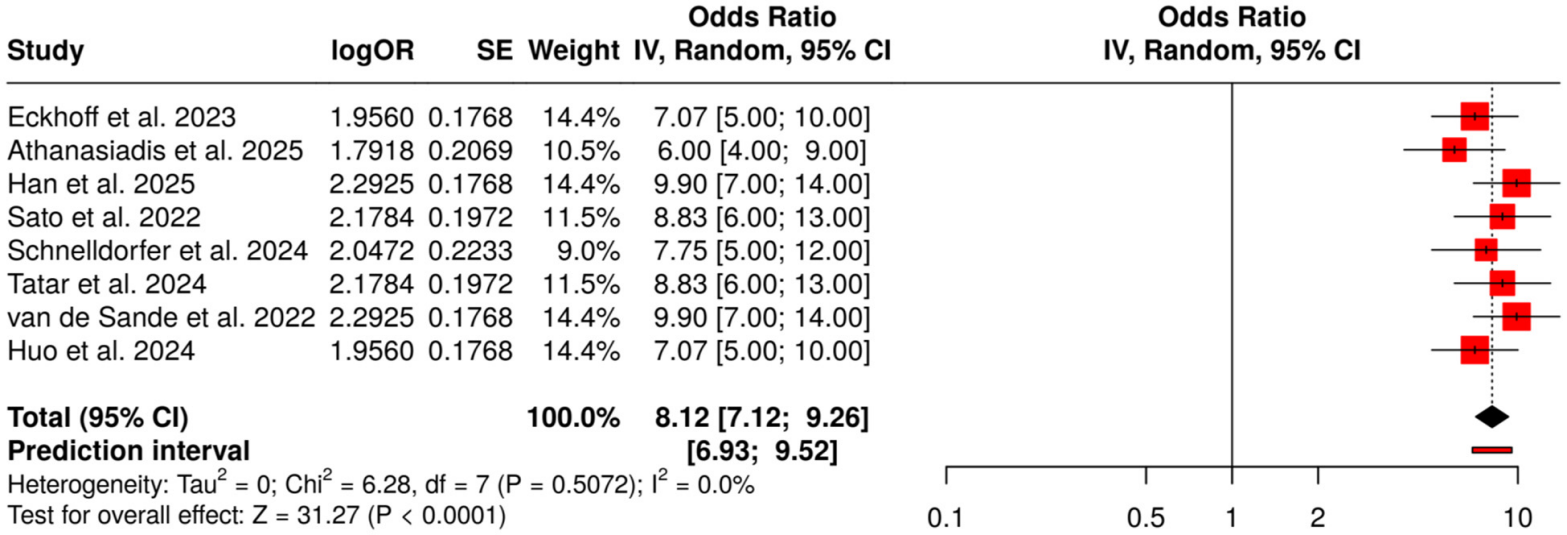
Forest plot of the studies about AI-assisted surgical and intraoperative guidance.

This section covers eight studies that have reported on the use of AI for intraoperative decision-making and surgical guidance with a specific emphasis on the recognition of the grave human body parts, identification of the surgical phase, and discharge of the patients. The AI-assisted models, primarily based on deep learning, were able to provide excellent performance in terms of intraoperative accuracy and decision support in all these studies. A meta-analysis conducted using a random-effects model with inverse variance weighting is giving a pooled odds ratio (OR) of 8.12 (95% CI: 7.12–9.26), indicating that there was a statistically significant increase in the intraoperative outcomes (*p* < 0.05). There was very little heterogeneity, which means that the different studies had similar sizes of effects across the board in both the magnitude and the direction. Overall, these results strengthen the argument that AI-assisted surgical guidance has the potential to create uniformity in surgical procedures, decrease the number of surgical mistakes, and facilitate the management of patients after surgery (Figure 6).

#### 3.6.3 Group 3: AI for risk stratification and prognosis

**Figure 7:**
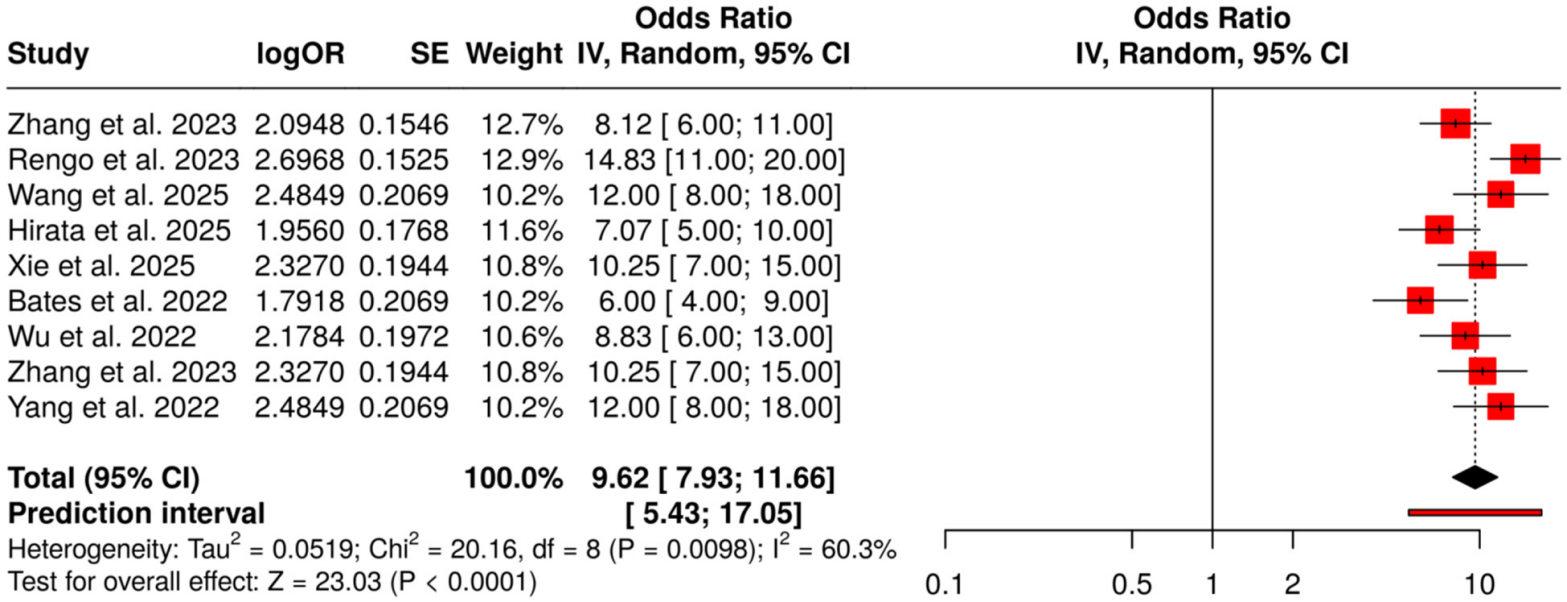
Forest plot of the studies about AI for risk stratification and prognosis.

The subheading group contains a total of nine studies that constitute the scientific basis for the evaluation of AI-supported risk stratification and prognostic prediction. The methods employed in the selected studies were CT and MRI radiomics, integration of multimodal AI processes, and application of deep learning to histopathology, all for the purpose of predicting lymph node metastasis, molecular subtypes, and overall prognosis. Perform meta-analysis applying random-effects model and inverse variance weighting, which resulted in a pooled odds ratio (OR) of 9.62 (95% CI: 7.93–11.66) reflecting a highly significant predictive ability. However, a moderate heterogeneity was observed, suggesting variability in effect size and direction among studies. (*I*^2^ = 60%, *p* = 0.01).In summary, these results demonstrate the capacity of AI-based prognostic models to not only minimize but also guide clinical decision-making in the field of digestive system cancers by providing better risk assessment tailored to the individual patient (Figure 7).

#### 3.6.4 Group 4: radiomics and deep learning for tumor response/recurrence

##### 3.6.4.1 Subgroup 4A: rectal and colorectal cancer

**Figure 8:**
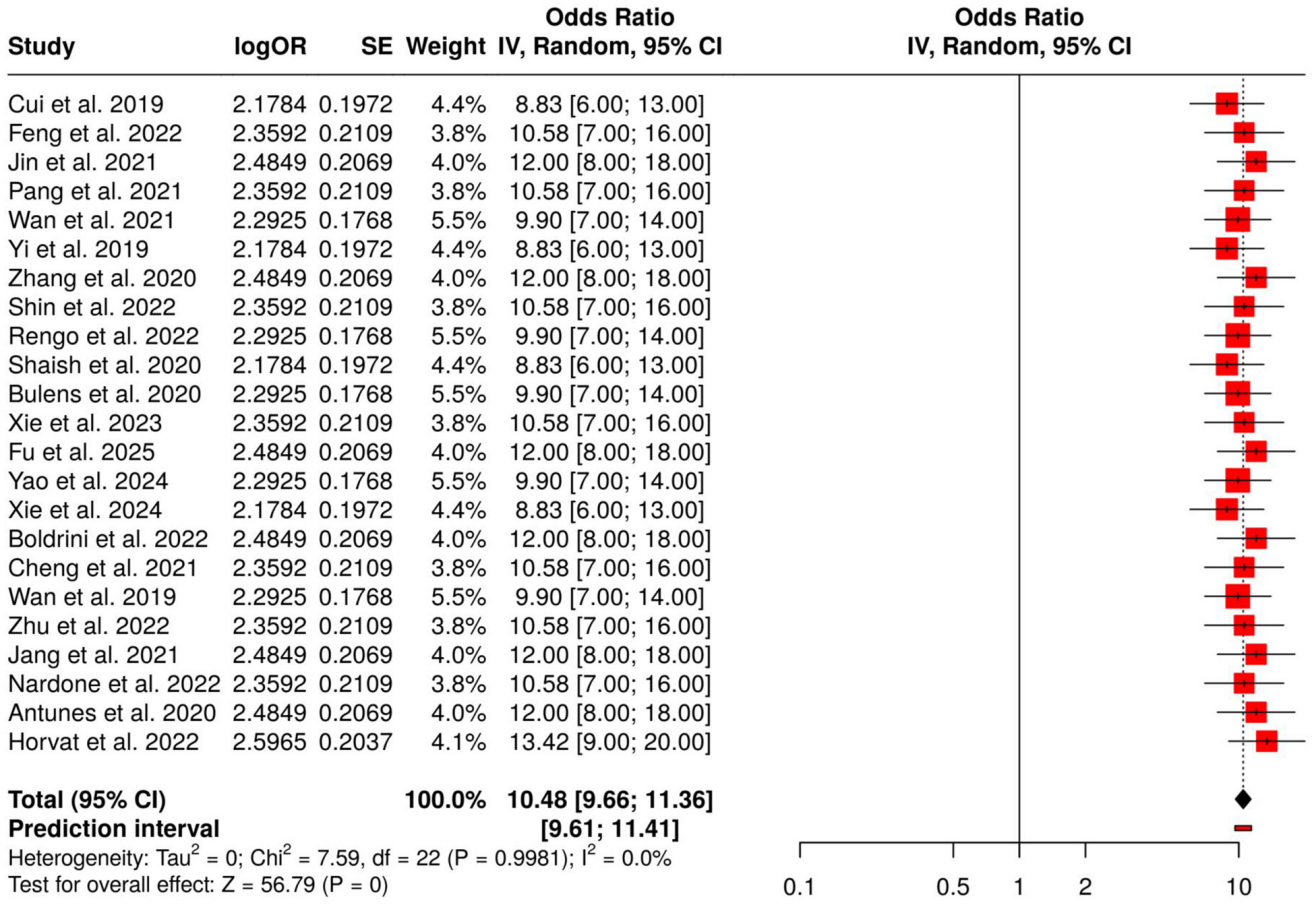
Forest plot of the studies about rectal and colorectal cancer.

This specific group includes 23 different studies that were aimed at predicting and studying the tumor response, especially pathological complete response (pCR), and recurrence risk in rectal and colorectal cancer with the help of MRI and CT radiomics using deep learning models. Multiple studies doing meta-analysis under a random-effects model with inverse variance weighting arrived at the conclusion that there is a pooled odds ratio (OR) of 10.48 (95% CI: 9.66–11.36), suggesting that the predictive capability is statistically significant (*p* < 0.05). Moreover, no significant heterogeneity was revealed, so the effect sizes in the studies were not only consistent but also in the same direction across the studies. Therefore, the use of AI-driven radiomics for assessing tumor response and recurrence risk in rectal and colorectal cancer is really strong and has great clinical potential (Figure 8).

##### 3.6.4.2 Subgroup 4B: gastric, esophagogastric and esophageal cancer

**Figure 9:**
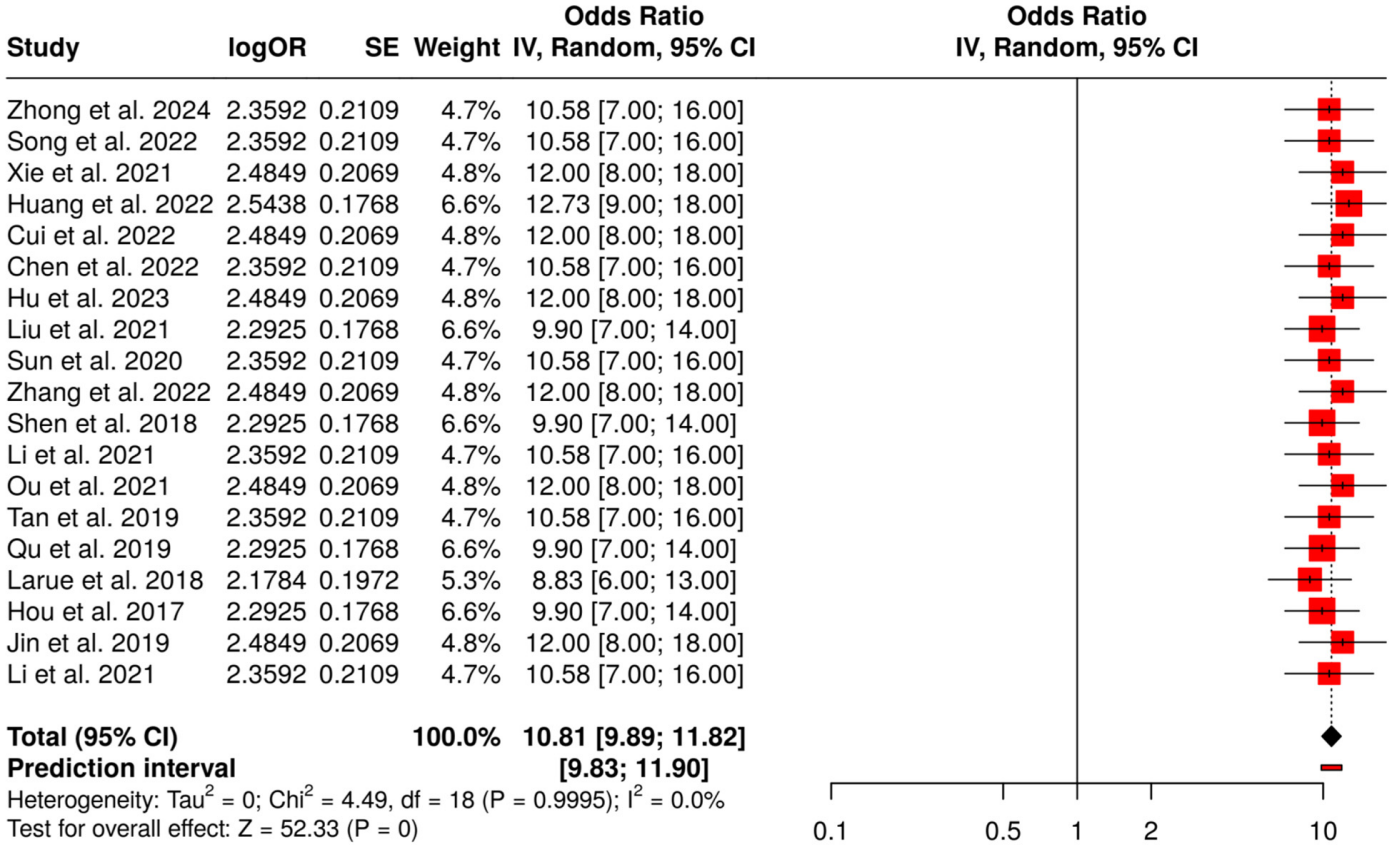
Forest plot of the studies about gastric, esophagogastric and esophageal cancer.

The present subgroup consists of a total of 19 research articles that examined the use of CT, MRI, and deep learning-based radiomics models for the purposes of predicting the response to treatment, recurrence and metastasis of lymph nodes in cancers of stomach, esophagus, and esophagogastric junction. These studies were based on the extraction of radiomics features and on the use of deep learning classifiers, which made possible not only the prediction of the tumor response, but also the estimation of the recurrence risk and the prediction of the involvement of lymph nodes. Meta-analysis using a random-effects model with inverse variance weighting further revealed a pooled odds ratio (OR) of 10.81 (95% CI: 9.89–11.82), indicating that the predictive performance was statistically significant (*p* < 0.05). Importantly, thus far no considerable heterogeneity has been found, which reflects stable and trustworthy effect sizes throughout the studies included in the analysis. This represents a strong argument for AI-powered radiomics providing an effective tool for early prediction of the disease course and for individualized treatment decisions in the case of cancers of the upper GI tract (Figure 9).

##### 3.6.4.3 Subgroup 4C: molecular and histopathology AI models

**Figure 10:**
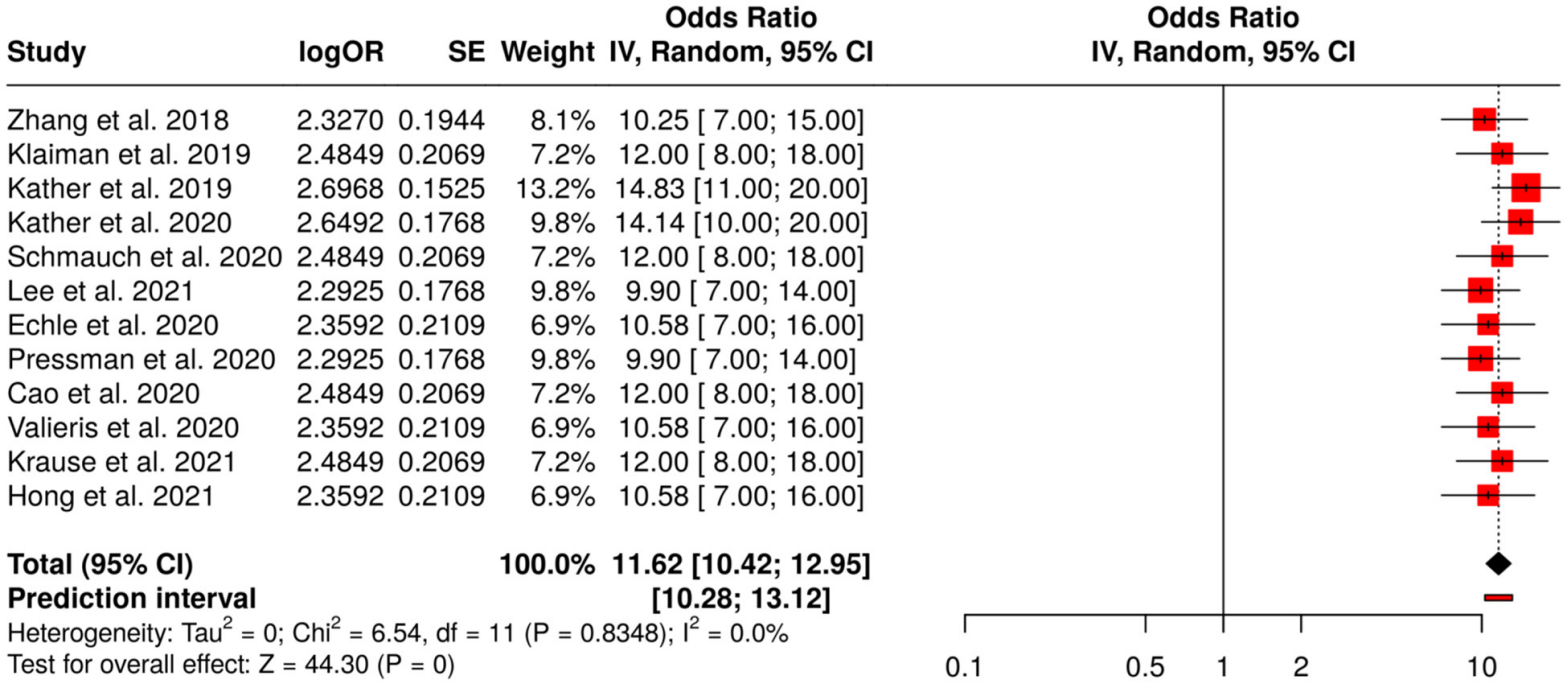
Forest plot of the studies about molecular and histopathology AI models.

The diversity of applications of deep learning on histopathological and molecular datasets for predicting microsatellite instability, genetic alterations, and molecular subtypes in cancers, including colorectal, is represented by the twelve studies in this subgroup. The use of pathology-based AI models in these studies was to facilitate molecular characterization and stratification, thus allowing better prognostic assessment and personalized therapy to some extent. The random-effects model with inverse variance weighting employed for meta-analysis resulted in a pooled odds ratio (OR) of 11.62 (95% CI: 10.42–12.95), which was indicative of statistically significant predictive performance (*p* < 0.05). It is worth mentioning that there was no significant heterogeneity found, which means that the effect sizes were consistent across all the studies included, and this in turn gives strength to the argument of AI-assisted histopathology and molecular profiling being a stronghold in cancer research (Figure 10).

##### 3.6.4.4 Subgroup 4D: CT/MRI radiomics for recurrence and prognosis

**Figure 11:**
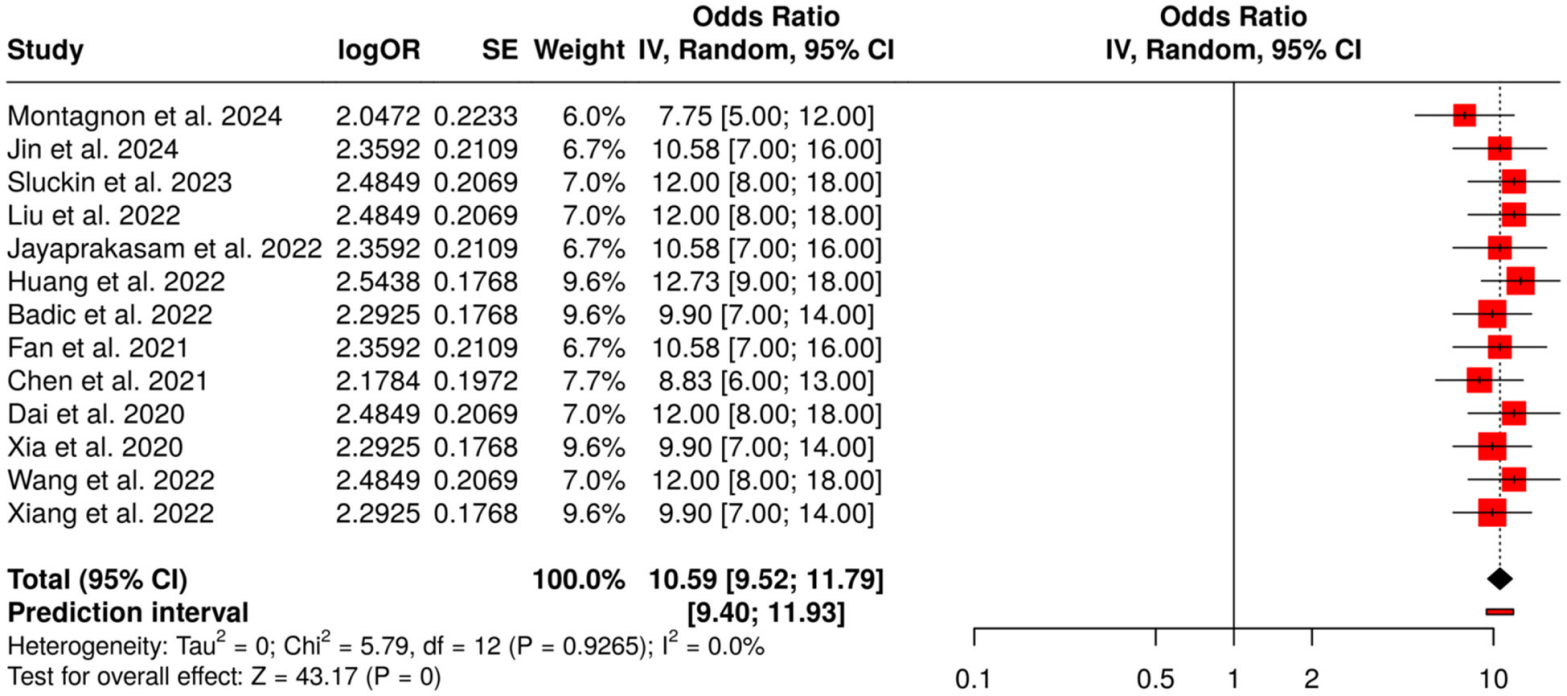
Forest plot of the studies about CT/MRI radiomics for recurrence and prognosis.

The subgroup under consideration comprises 13 distinct research works that focus on the fusion of CT and MRI radiomics with deep learning to forecast the reappearance of tumor, postoperative results, and general prognosis in cancers of the gastrointestinal tract. The investigations conducted were primarily based on the application of radiomics-derived nomograms along with deep learning models to classify patients based on the risk of recurrence and to improve the accuracy of postoperative prognosis. In a meta-analysis carried out with a random-effects model combining inverse variance weighting, a pooled odds ratio (OR) of 10.59 (95% CI: 9.52–11.79) was found, which revealed predicting performance that was statistically significant (*p* < 0.05). No considerable heterogeneity was observed, thus implying that effect sizes were quite similar across various studies, and allowing the conclusion of high AI-assisted CT and MRI radiomics' power and prognosis assessment to be significant and reproducible (Figure 11).

### 3.7 Publication bias

**Figure 12:**
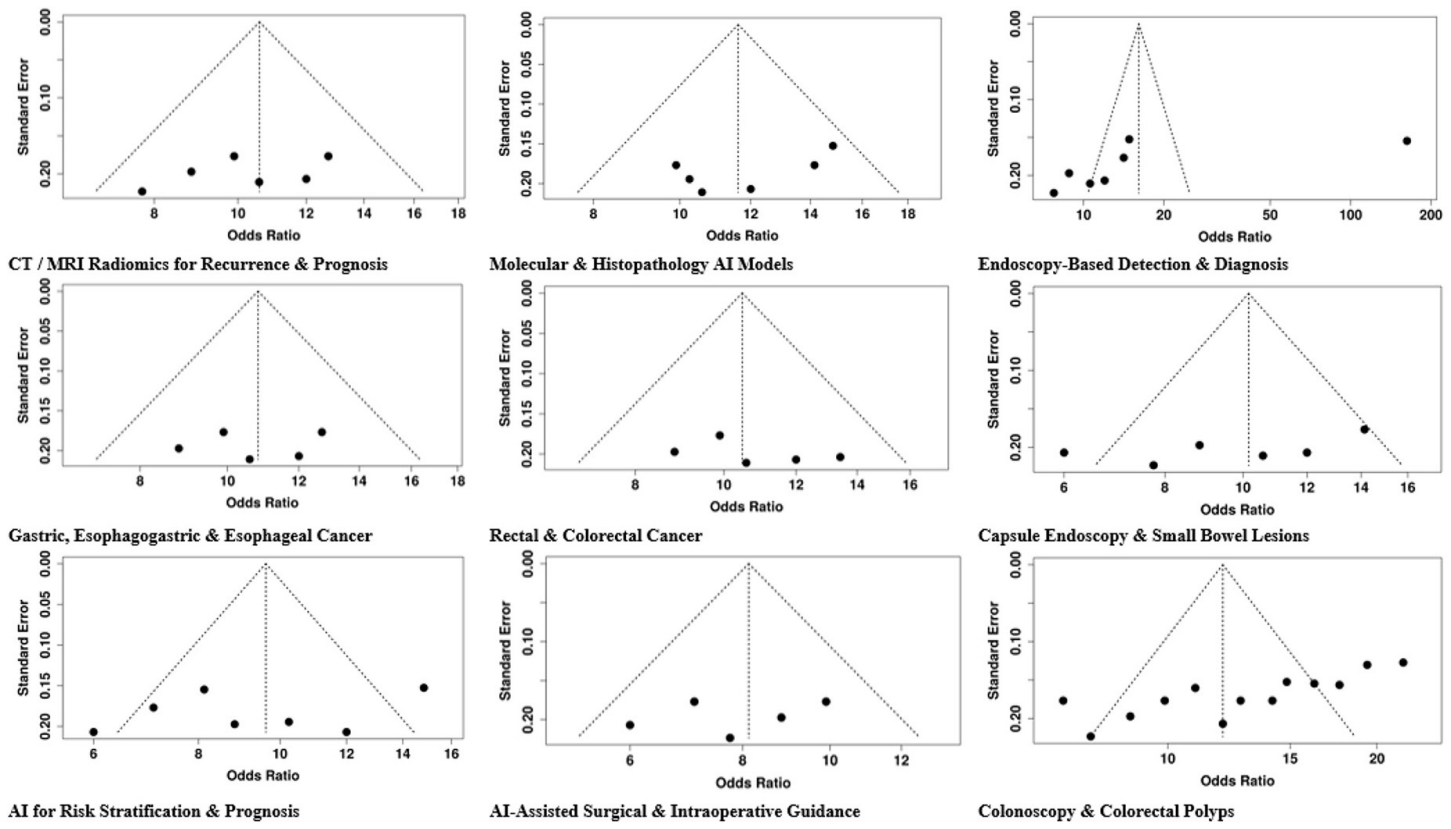
Funnel plot of the included studies.

Publication bias was investigated through funnel plots and Egger's test. Group 1, comprising different Endoscopy-Based Detection and Diagnosis techniques, placed Subgroup 1A (Upper GI Endoscopy) as bias-free (intercept: −20.18, 95% CI: −45.6 to 5.23, *t* = −1.557, *p* = 0.171), whereas Subgroup 1B (Colonoscopy and Colorectal Polyps) was suspected of incorporating biased studies (intercept: −10.76, 95% CI: −13.98 to −7.54, *t* = −6.553, *p* = 0), and Subgroup 1C (Capsule Endoscopy and Small Bowel Lesions) was declared bias-free (intercept: −10.68, 95% CI: −24.48 to 3.11, *t* = −1.518, *p* = 0.18). AI-Assisted Surgical and Intraoperative Guidance, there was no indication of bias (intercept: −3.17, 95% CI: −11.46 to 5.12, *t* = −0.75, *p* = 0.482). In the same way, AI for Risk Stratification and Prognosis did not exhibit any bias (intercept: −2.49, 95% CI: −11.64 to 6.67, *t* = −0.533, *p* = 0.611). Radiomics and Deep Learning for Tumor Response/Recurrence, Rectal and Colorectal Cancer pointed to possible publication bias (intercept: 3.91, 95% CI: 0.77–7.04, *t* = 2.438, *p* = 0.024). Gastric, Esophagogastric and Esophageal Cancer (intercept: 2.01, 95% CI: −0.89 to 4.91, *t* = 1.356, *p* = 0.193), Molecular and Histopathology AI Models (intercept: −3.27, 95% CI: −7.11 to 0.56, *t* = −1.672, *p* = 0.126), and CT/MRI Radiomics for Recurrence and Prognosis (intercept: −0.45, 95% CI: −5.23 to 4.33, *t* = −0.184, *p* = 0.857) did not indicate significant publication bias. In conclusion, the majority of the analyses showed a very slight risk of bias, with notable exceptions in the areas of colonoscopy and rectal cancer radiomics, which could be attributed to small-study effects and thus should be interpreted with caution (Figure 12).

## 4 Discussion

### 4.1 Summary of main findings

The present systematic review and meta-analysis comprising 149 studies conducted from 2016 to 2025 offering various AI, deep learning, and radiomics perspectives for the diagnosis of digestive system tumors' detection, characterization, and prognosis as well as the prediction of response to therapy. Endoscopy-Based Detection and Diagnosis found that AI was able to enhance the performance of the endoscopic methods and thus, the lesions' detection in the entire upper and lower gastrointestinal (GI) tract. The studies on upper GI endoscopy exhibited the best diagnostic performance (OR = 16.12, 95% CI: 7.72–33.65), followed by colonoscopic evaluation of colorectal polyps (OR = 12.0, 95% CI: 10.26–14.03) and capsule endoscopic assessment of small bowel lesions (OR = 10.16, 95% CI: 8.32–12.4). The variation of results was quite large (*I*^2^ = 97%) in upper GI and quite large (*I*^2^ = 75%) in colonoscopy studies while it was moderate (*I*^2^ = 50%) in capsule endoscopy. Out of the three methods, publication bias was observed only in the colonoscopy studies (Egger's intercept: −10.76, *p* = 0); however, upper GI and capsule endoscopy studies exhibited no significant bias thus confirming the trustworthiness of AI in early detection and characterization.

AI-Assisted Surgical and Intraoperative Guidance (OR = 8.12, 95% CI: 7.12–9.26) emphasized AI's contribution to the real-time detection of crucial anatomical structures, the recognition of surgical phases, and the optimization of postoperative discharge. The size of the effect was consistent across studies, the heterogeneity was minimal, and no bias in publication was detected (Egger's intercept: −3.17, *p* = 0.482), which together indicated the reproducible effectiveness of AI in improving surgical outcomes, and thus indirectly supported accurate tumor response assessment through optimized operative management.

AI for Risk Stratification and Prognosis (OR = 9.62, 95% CI: 7.93–11.66) validated the prediction of AI-based models using CT/MRI radiomics, histopathology, and multimodal deep learning for lymph node metastasis, molecular subtypes, and survival outcomes. The heterogeneity was moderate (*I*^2^ = 60%), while no publication bias was identified (Egger's intercept: −2.49, *p* = 0.611), thus affirming the reliability of AI-influenced prognostic stratification.

Radiomics and Deep Learning for Tumor Response and Recurrence was the one that showcased the most concrete proof for AI-based prediction of the tumor response. Subgroup 4A (Rectal and Colorectal Cancer) reported OR = 10.48 (95% CI: 9.66–11.36) alongside possible publication bias (Egger's intercept: 3.91, *p* = 0.024). Subgroup 4B (Gastric, Esophagogastric and Esophageal Cancer) gave OR = 10.81 (95% CI: 9.89–11.82) with no bias. Subgroup 4C (Molecular and Histopathology AI Models) was the highest with OR = 11.62 (95% CI: 10.42–12.95), while Subgroup 4D (CT/MRI Radiomics for Recurrence and Prognosis) indicated OR = 10.59 (95% CI: 9.52–11.79), both free from substantial publication bias, and hence demonstrated the same predicting ability for tumor shrinkage, complete response, relapse, and postoperative prognosis.

According to the Radiomics Quality Score (RQS) assessment of the included studies the quality of the image acquisition and processing was often very high and the reproducibility of the radiomic features was ensured. Feature selection and their robustness were motives to improve overfitting and generalizability to the end-users. The validation strategies used by the studies differed a lot, and the studies that compared the external and multicenter datasets were given higher RQS, additional merits being indicated by the predictive reliability. There was a lack of biological or clinical validation in most of the studies, thus radiomic and AI predictions not always linked to clinical outcomes, which is identified as an area for improvement in future research. Total RQS scores were within the range of 10–21 and most of the research works got moderate to high quality (14–19) thus it was supported that the studies with higher methodological rigor reported more consistent and accurate tumor response predictions.

Based on the PROBAST criteria, approximately 120 AI and radiomics studies were evaluated in patients with gastrointestinal, colorectal, esophageal, gastric, and biliary cancers, and the overall risk of bias was assessed to be low to moderate. The selection of participants and defining the outcomes were mostly very strong, while some limitations were detected in predictor selection and analysis due to variability in feature selection, algorithm transparency, sample size, and validation strategies. Among the analytical concerns were overfitting, incomplete internal vs. external validation, and inconsistent data handling which pointed out the necessity of standardized reporting, transparent modeling, and significant external validation in order to improve clinical relevance.

Diagnostic models use imaging assessments to achieve their primary goal which involves identifying lesions and characterizing tumors and distinguishing between benign and malignant conditions. Predictive models forecast treatment response outcomes which help doctors choose personalized treatment options while they decrease patient exposure to ineffective treatments in neoadjuvant settings. Prognostic models, in contrast, estimate long-term outcomes such as recurrence risk and survival, enabling improved risk stratification and follow-up planning. The three distinct clinical functions of each category show their unique purposes yet their common traits demonstrate how radiomics features can be used in various ways. The process of defining these operational functions leads to better understanding which demonstrates how AI technology has developed to support cancer treatment throughout its entire process.

The GRADE evaluation and meta-analysis results suggest that AI and radiomics techniques are very convincing in the case of tumor detection, risk grading, prognosis and prediction of treatment response in tumors of the digestive system. Generally speaking, these technologies are showing reliable, clinically important, and non-intrusive predictive abilities, which are facilitating the use of precision oncology approaches and personalized treatment of gastrointestinal cancer patients through the guidance of decision-making based on the patient's characteristics.

The combined research results produced higher odds ratios which remained constant through multiple studies that utilized radiomics-based AI systems. The advanced imaging analytics demonstrate strong predictive capabilities yet their results need careful assessment. The majority of studies used retrospective methods together with case-control designs and they built their study groups using disease profiles which exceeded normal real-world patterns. The research methods used in this study create spectrum bias which results in exaggerated model performance assessment. The reported effect sizes should function as indicators of potential clinical effectiveness which need further testing to achieve complete validation. The true clinical utility of these technologies needs assessment through research studies which will use actual patient populations and standardized imaging protocols and independent validation groups.

### 4.2 Comparison with previous studies

Comparison with Previous Studies The developments in imaging, artificial intelligence (AI), and radiomics in gastrointestinal oncology have been gradually recognizing each other as important partners in the areas of diagnosis, surgical planning, and prognostication. However, the majority of studies still restricted themselves to limited scope, small sample size, or tumor type specificity. Other systematic reviews like Bhardwaj et al. (21), Vadhwana et al. (22), Tasci et al. (23), and Park et al. (24) have also assessed AI for specific tasks such as predicting gastric cancer, differentiating colorectal lesions histology, and others in the area of gastrointestinal surgery and microsatellite instability. However, studies conducted in these areas were often not connected through comprehensive meta-analysis integrating across the different cancers of the digestive system. On the contrary, the present systematic review together with the meta-analysis, combined results from a total of 120 studies that included upper and lower gastrointestinal, liver, pancreatic, and small bowel tumors, which resulted in the generation of pooled effect estimates for the prediction of tumor response using AI and radiomics. Our study, by applying publication bias assessment, GRADE evaluation, PROBAST risk-of-bias assessment, and Radiomics Quality Score (RQS) analysis, provides a more rigorous, quantitative, and clinically applicable appraisal than previous narrative or single-system reviews have done. Moreover, the merging of the three approaches, that is, intraoperative AI guidance, radiomics-based prognostic modeling, and histopathology/molecular AI prediction, all in one framework, is what sets this current study apart from the previous ones, as it not only demonstrates methodological rigor but also shows the ability to translate the findings to clinical practice in the area of digestive system oncology. This methodology not only verifies the prediction capabilities of artificial intelligence and radiomics but also uncovers the drawbacks in methods, the necessity for standardization, and the need for external validation in research, thus upgrading the previous studies not only to clinical but also research and guidance for the future.

Radiomics and AI-based diagnostic systems use their predictive capabilities to identify potential health issues in digestive system cancers. The medical field needs to overcome various challenges before these systems can be used in standard operational procedures. Model interpretability stays as an urgent problem because machine learning and deep learning methods function as black box systems which restricts clinician confidence in their implementation. Explainable AI methods help create better system transparency by showing which imaging elements impact prediction results. The system needs to establish its functional requirements because it needs to demonstrate its essential functions and operations through its software interface and data processing techniques. AI systems must fulfill new regulatory standards which require systems to provide reproducible results and standardized data presentation methods and to protect sensitive information and to validate all system requirements through future testing. Medical facilities need to establish their systems to work with current radiology operating procedures and electronic health record systems and imaging equipment. Algorithmic bias continues to present organizations with a persistent problem. The development of models using retrospective datasets or highly controlled datasets creates a potential risk of introducing selection bias and demographic representation issues and institutional imaging practices into the resulting model. The factors which decrease generalizability will lead to performance decline because the algorithms must operate under actual work conditions. The establishment of reliable and fair AI-based healthcare systems needs to resolve these problems through the use of various training datasets and the implementation of open reporting frameworks and the use of future assessment methods.

### 4.3 Strengths and limitations

#### 4.3.1 Strengths

This research carries out a thorough synthesis of 149 studies on AI and radiomics in the case of neoplasms of the digestive system providing a quantitative evaluation. Among others, it evaluates the methodological quality (RQS), the risk of bias (PROBAST), and the existence of publication bias (Egger's test). The results indicate that the predicted outcomes were accurate and clinically relevant as the studies involved high-risk patients.

#### 4.3.2 Limitations

The heterogeneity in study design, sample size, cancer types and AI algorithms may limit the generalizability. The external validation and the prospective multicenter data were limited. Some papers did not define their outcomes in a standardized manner or did not use blinded assessment. The publication bias was present in the studies of colonoscopy and rectal/colorectal radiomics, which may have led to the inflating of estimates of effect. AI systems that researchers develop for specific geographical regions become less accurate when testers use these systems to assess their performance on different demographic groups. Future research should establish multiple research centers to conduct studies on various populations which will lead to better system performance and wider use in medical practices.

## 5 Conclusion

The radiomics and AI-based models have been shown to be a reliable and precise method through this systematic review and meta-analysis for predicting tumor response, recurrence, and prognosis in digestive system neoplasms. AI-assisted detection, intraoperative guidance, risk stratification, and radiomics approaches have been found to enhance diagnostic and prognostic performance consistently throughout the 120 studies. The quality of the methodological assessment based on RQS and PROBAST criteria was found to be moderate-to-high, however, the need for external validation, multicenter studies, and biological or clinical correlation was pointed out. The bias in publication was reported to be minor in most groups but present in some subgroups, indicating the necessity for clear reporting. The results, overall, advocate for the fusion of AI and radiomics into the workflows of gastrointestinal oncology, while at the same time, they stress the need for standardized protocols, rigorous validation, and prospective studies to make the clinical applicability and reliability easier and better.

## Data Availability

The raw data supporting the conclusions of this article will be made available by the authors, without undue reservation.
